# Mapping Biodiversity Conservation Priorities for Protected Areas for Spatial Optimization: A Case Study in the Songnen Plain, China

**DOI:** 10.1002/ece3.70516

**Published:** 2024-11-05

**Authors:** Qiaoyun Sun, Jianqi Yu, Yingran Zeng, Yifang Gai, Jia Wang, Yujun Zhang

**Affiliations:** ^1^ School of Architecture and Urban Planning Shenzhen University Shenzhen China; ^2^ School of Landscape Architecture Beijing University of Agriculture Beijing China; ^3^ Shanghai Key Laboratory of Urban Design and Urban Science NYU Shanghai Shanghai China; ^4^ School of Ecological and Environmental Sciences East China Normal University Shanghai China; ^5^ Beijing Institute of Landscape and Traditional Architectural Design and Research co Ltd Beijing China; ^6^ Institute of Spatial Planning Soochow University Suzhou China; ^7^ School of Landscape Architecture Beijing Forestry University Beijing China

**Keywords:** biodiversity hotspots, conservation gaps, conservation priorities, declines in biodiversity, protected areas, the Songnen Plain

## Abstract

The decline in biodiversity poses a serious threat to natural ecosystems and has become one of the most pressing global environmental issues. Establishing conservation priorities for protected areas (PAs) is one of the most direct and effective biodiversity conservation measures. However, conservation gaps arise as a result of existing problems in spatial layout of PAs, including overlapping protection scopes, artificial fragmentation of natural ecological regions, as well as “over‐protection” and “over‐exploitation.” To optimize the spatial layout of PAs and improve the efficiency of biodiversity conservation, we employed the Habitat Quality module of the Integrated Assessment of Ecosystem Services and Tradeoffs (InVEST) model and the Maximum Entropy (MaxEnt) model to assess the PAs in the Songnen Plain, China. The combined model (MaxEnt‐InVEST) revealed that the conservation priorities for PAs in the Songnen Plain occupied a total area of 14,764.14 km^2^ (10.24% of the total area of the Songnen Plain). The conservation priorities outside PAs occupied a total area of 7858.45 km^2^ (5.45% of the total area of the Songnen Plain) and were primarily distributed in the northeastern, central, and southwestern regions of the Songnen Plain. This indicated that existing PAs did not offer adequate protection for local biodiversity. The consistency of our combined modeling framework was 72.11%, which enabled a more accurate assessment of biodiversity hotspots and respects the land uses of the Songnen Plain. In addition, the modeling framework successfully created maps of conservation gaps of biodiversity hotspots based on actual species distribution data and considers current land uses. Our study was aimed at optimizing the spatial conservation efficiency of the Songnen Plain by assessing the conservation gaps in the Songnen Plain. It could provide a reference for the future development of a PA system centering on national parks.

## Graphical abstract

The combined model (MaxEnt‐InVEST) reveals that the conservation priorities for PAs in the Songnen Plain occupy a total area of 14,764.14 km^2^ (10.24% of the total area of the Songnen Plain). This indicates that existing PAs do not offer adequate protection for local biodiversity. The consistency of our combined modeling framework is 72.11%, which realizes a more accurate assessment of biodiversity hotspots and respects the land uses of the Songnen Plain.

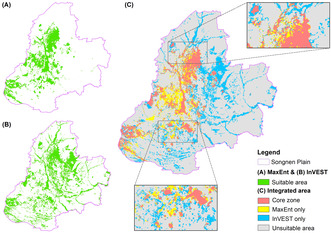

## 1 Introduction

The loss of biodiversity has become one of the major global environmental issues (Cardinale et al. 2012; Cowie, Bouchet, and Fontaine 2022; IUCN 2022). The primary drivers of biodiversity loss are habitat destruction and fragmentation due to anthropogenic disturbance and land‐use change (Newbold et al. 2016). Currently, in highly urbanized plain areas, long‐term anthropogenic activities have caused a significant reduction in natural habitat, leading to increased landscape fragmentation (Zhang, Zeng, and Namaiti 2023). Therefore, establishing an effective protected area (PA) system in plain areas to reduce habitat loss and fragmentation is crucial in alleviating the decline of biodiversity (Le Saout et al. 2013; Eichenwald, Evans, and Malcom 2020).

However, as PA planning is a complex process (Cui and Wang 2000), its spatial layout and site selection involve various aspects, such as conservation objectives, habitat quality, and conservation strategies (Moilanen and Arponen 2011; Kujala et al. 2013; Cao et al. 2020). The PA system in China faces a series of challenges, including overlapping PA boundaries and the artificial fragmentation of natural ecological zones, which leads to the coexistence of “over‐protection” and “overuse.” This issue is particularly prominent in plain areas (Xu et al. 2019). Establishing a PA system centered around national parks, along with a streamlined, efficient, and unified management framework, represents a crucial institutional reform to address the problems of “over‐protection” and “over‐utilization” (Zhong et al. 2016; Cao et al. 2020). In the reform of the new PAs system, there is an urgent need for a detailed analysis of the protection gap using methods tailored to the characteristics of plain areas (Xu et al. 2019).

Gap analysis is based on high‐quality environmental variables and ecosystem condition data to plan the layout and location of PAs (Catullo et al. 2008). It is particularly useful in areas where biodiversity data are scarce (Sharafi et al. 2012). Gap analysis begins by modeling conservation priorities and then comparing these results with existing PA coverage. One method involves applying an empirical assessment strategy that follows standardized procedures established by the International Union for Conservation of Nature (IUCN). This includes a “red list” of threatened species, which is now an internationally recognized method for identifying protected species (Zhang and Ma 2008; Hayward 2011). Another approach is to use data from museums or field surveys alongside species distribution models (SDMs) to predict priority areas for biodiversity conservation. This method is essential for protecting the biodiversity of endemic or endangered species (Myers et al. 2000; Ceballos and Ehrlich 2006; Pascual et al. 2011). Based on different algorithm rules and prediction purposes, various predictive models have been derived from SDMs, including the Maximum Entropy (MaxEnt) model (Phillips, Anderson, and Schapire 2006), CLIMEX model (Shabani, Kumar, and Taylor 2012), Ecological Niche Factor Analysis (ENFA) (Wiens et al. 2009), and Genetic Algorithm for Ruleset Prediction (GARP) (Hernandez et al. 2006). However, the accuracy of assessments aimed at identifying conservation priority areas and constructing conservation networks is hindered by insufficient data for most threatened species (Collen et al. 2008).

To overcome the lack of information on real species sites, different methods have been adopted to map biodiversity priorities, such as habitat quality and net primary productivity (NPP) (Ma et al. 2019). Although these methods are reasonable in the absence of species data, they may be inadequate as a foundation for conservation management (Han et al. 2019). For this reason, there is a need to explore a new approach that allows for a more accurate and rational identification of conservation gap areas for use in conservation networks. The MaxEnt model has been widely used for distribution modeling of potential species (Phillips, Anderson, and Schapire 2006; Huang et al. 2020; Zhao, Xiao et al. 2022). Despite small sample sizes, MaxEnt exhibits a considerable level of robustness. However, in most instances, the actual land‐use status is overlooked when using the MaxEnt model, potentially resulting in predictions that are better than the real case (Hughes 2017; Zhao, Xiao et al. 2022). In simpler terms, a complex model might be overfitting. Therefore, the use of the Integrated Valuation of Ecosystem Services and Tradeoffs (InVEST) model can enhance accuracy in predictions, correcting overestimations and showing the original habitat quality in terms of land‐use vulnerability (Babbar et al. 2021; Huang, Qian, and Cao 2022). In addition, collaborative protection of land‐use types and species diversity hotspots is one of the most cost‐effective and efficient conservation methods for spatial planning in PAs (Watson et al. 2020). This approach can analyze the relationship between urbanization and existing biodiversity in the area (Zhang, Zeng, and Namaiti 2023).

Recent studies have increasingly explored the integration of various model analysis methods to enhance the accuracy of identifying conservation priority areas. For instance, combining MaxEnt with Circuitscape has been used to assess the suitability of bird conservation areas (Barik, Saha, and Mazumdar 2021). Additionally, integrating MaxEnt with Zonation has helped determine the spatial distribution of priority PAs for multiple species in Xiamen, China (Huang, Qian, and Cao 2022). Moreover, a combination of MaxEnt and InVEST has been applied to analyze plant conservation priority areas in the Xishuangbanna tropical area (Huang et al. 2020). These integrated approaches have indeed improved the precision of conservation priority identification. However, it remains uncertain whether these methods are equally effective in plain areas with high levels of human activity and minimal environmental variability. Further research is needed to assess the applicability of these comprehensive models in such contexts.

The Songnen Plain plays a crucial ecological role in Northeast China, serving as a vital area for flood control, water storage, sand fixation and soil consolidation in the region (Lv et al. 1998; Zhao, Wang et al. 2022). In addition, the Songnen Plain has an intact and pristine wetland ecosystem in Northern China (Yang and Song 2021), which is home to key nationally protected species (Wu et al. 2015). Due to long‐term uncontrolled development and construction, the biological habitat (e.g., wetlands) has been damaged to a certain extent in the Songnen Plain (Chu et al. 2022). Despite the establishment of certain PAs, their current efficacy in safeguarding the habitats of endangered species and ecosystem services in the Songnen Plain is insufficient (Lu et al. 2015; Yang and Song 2021). Protection levels and management efforts among conservation agencies remain inconsistent (Xu et al. 2019). Moreover, weak connectivity between PAs leads to islanding and fragmentation of waterfowl habitats (Wang et al. 2011; Lu et al. 2015). Therefore, the optimization of the spatial layout of the Songnen Plain PAs should be promoted as soon as possible.

Our study proposed a feasible and applicable framework for identifying conservation gaps in plain ecological areas by integrating MaxEnt and InVEST models. We centered on identifying the conservation gaps between existing PAs and conservation priority areas. Additionally, we evaluated potential methods of enhancing the protection of species' natural habitats and regional biodiversity patterns in plain areas characterized by high levels of urbanization. Therefore, the overall objective of the current study was to establish a conservation model framework, with the Songnen Plain serving as a case study. The framework is expected to assist policymakers in evaluating the efficiency of existing PAs and devising a new PA system centered on national parks. The specific objectives of the study are to: (1) map biodiversity hotspot areas in the Songnen Plain; (2) assess conservation gaps in existing PAs; and (3) prioritize a PA system based on the hotspot areas identified through modeling.

## 2 Materials and Methods

### 2.1 Study Area

**FIGURE 1:**
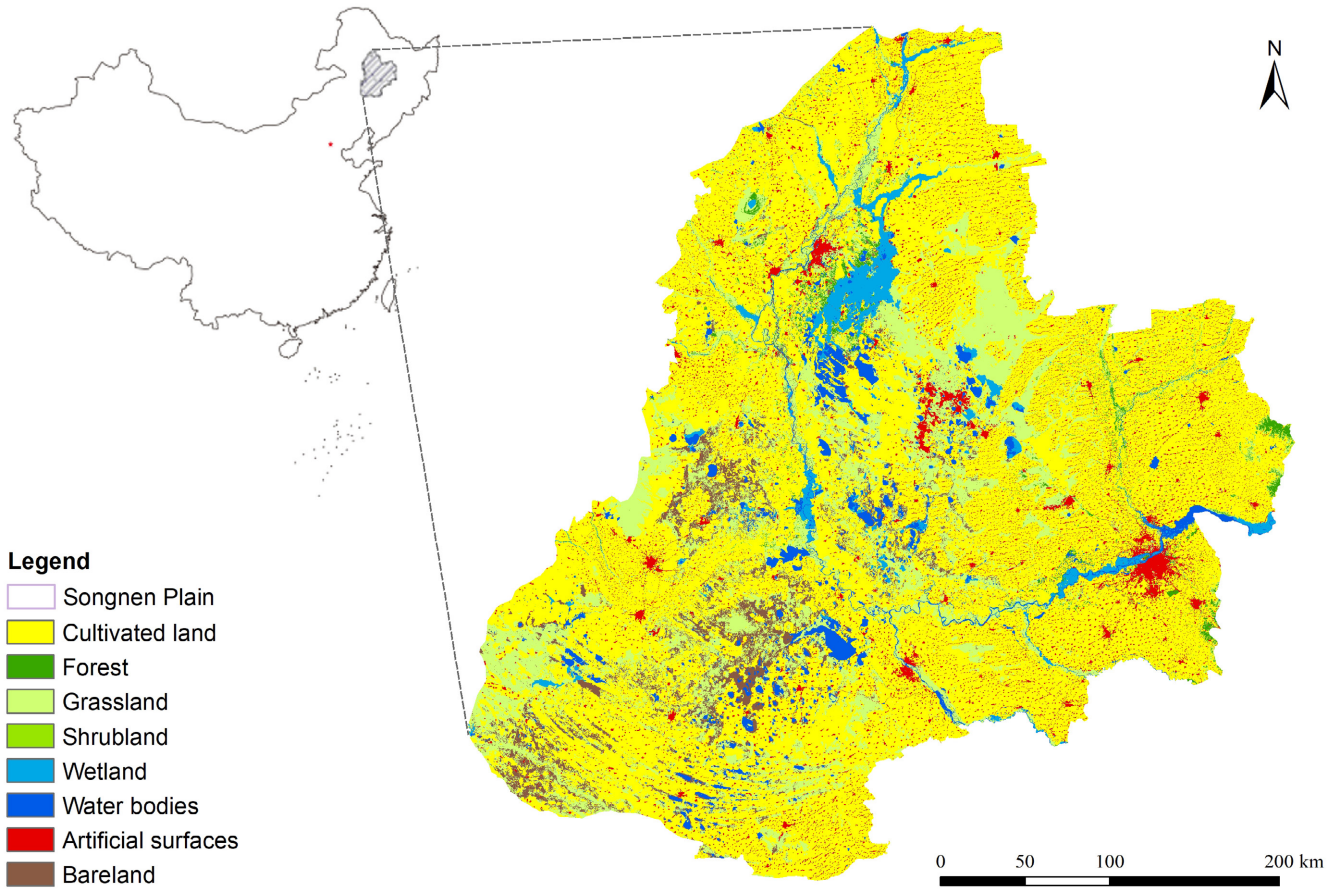
Study area and land‐use status.

The Songnen Plain (43°59′22″–48°33′49″ N, 121°34′12″–127°41′56″ E) is located in the central‐western part of Northeast China and is one of the largest plains in China (Figure 1), forming the Northeast Plain together with the Sanjiang Plain and the Liaohe Plain (Liu 2010). The region is located in the central area between the Da Hinggan Range, the Xiao Hinggan Range, the Changbai Mountains, and the Songliao Basin and has an overall rhombus‐shaped geospatial configuration (Liu 2010). Through the cross‐reference of the terrain, watershed divisions and administrative divisions in Northeast China, the approximate scope of the Songnen Plain is finally determined as the research area, with a total area of 14.42 × 10^4^ km^2^ (Sun et al. 2022). The major climate types in the study area include temperate continental semi‐humid and semiarid monsoon climates (Chu et al. 2022). The land‐use types in the Songnen Plain are mainly field land, followed by water and grassland (Qiu et al. 2017). The abundance of natural resources transforms the region into a breeding ground and habitat for numerous waterfowl species, including the red‐crowned crane, oriental white stork, and wild duck (Zhou et al. 2013; Yang and Song 2021). According to the statistical calculations from the Comprehensive Scientific Investigation of seven national nature reserves, there are 1067 species of wild plants and 513 species of wild animals, including 349 species of birds, accounting for 24.15% of China's birds species (Table S1). Of these, there are 120 species of key protected species, including 10 species of seed plants (0.96% of the total), 7 species of mammals (12.07% of the total), and 103 species of birds (29.51% of the total).

However, with the acceleration of the urbanization process, land‐use changes have led to the destruction of numerous natural habitats (Chu et al. 2022). In addition to agricultural encroachment, regional urbanization and industrialization also largely removed substantial portions of natural land cover (Wang et al. 2011; Mao et al. 2019). Thus, the spatial layout of the Songnen Plain PA system should take into account potential land‐use changes in the future and habitat conservation.

### 2.2 Methodological Framework

**FIGURE 2:**
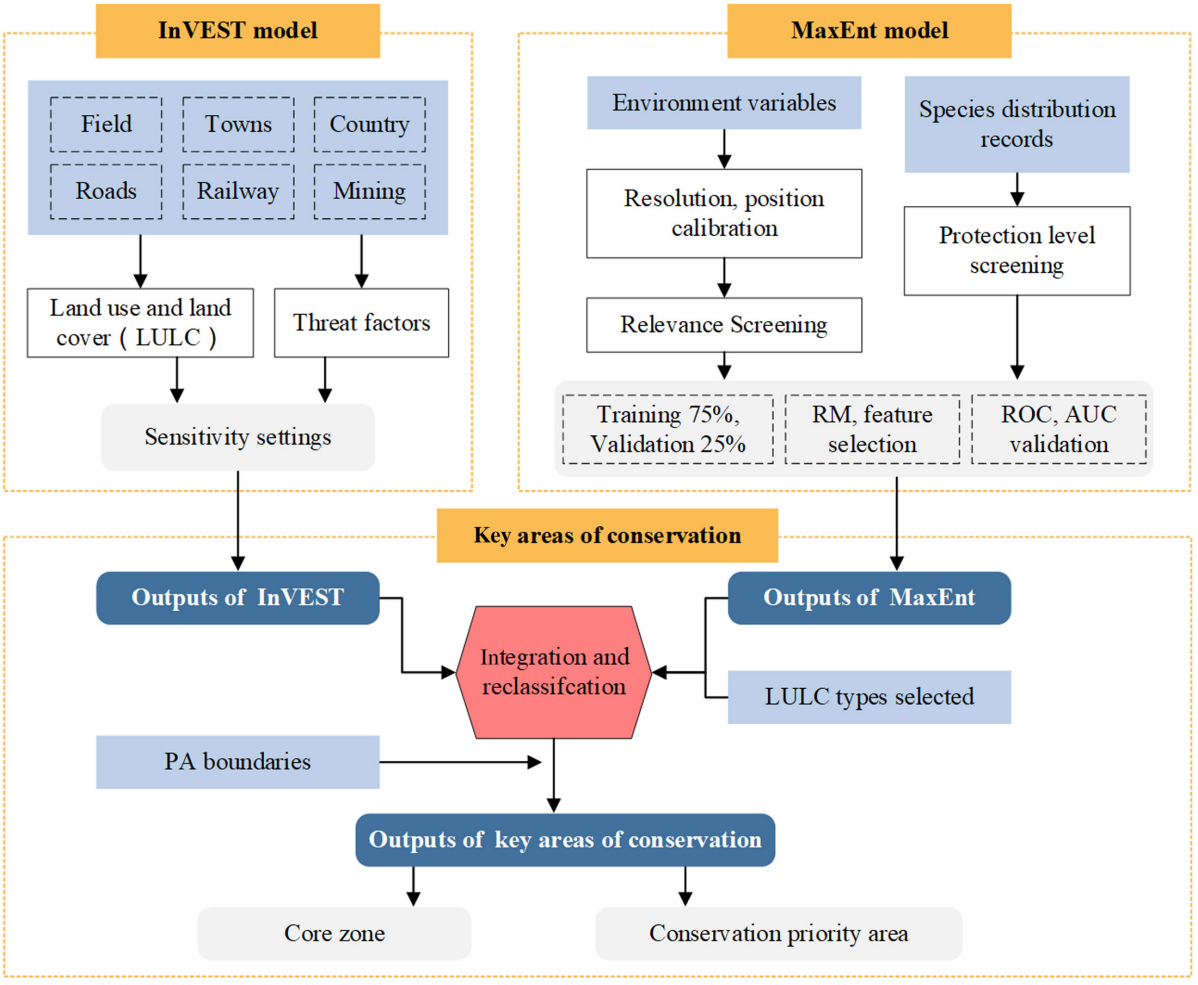
Flowchart of the methodological workflow in this study.

A flowchart was devised to summarize the entire methodology (Figure 2). The study applied two models to predict biodiversity hotspots in the Songnen Plain and to assess the impact of land‐use change on biodiversity habitat quality. The two models were combined to complement each other. First, the MaxEnt model was applied to explore the prediction of spatial species distribution patterns using environmental variables and species records. Second, the InVEST model was applied to assess the sensitivity of habitats to land‐use change in the Songnen Plain. Finally, the study results were applied to analyze ecological conservation gaps. All data were analyzed using ArcGIS 10.2 (ESRI Inc., Redlands, CA, USA).

### 2.3 MaxEnt Model Prediction

#### 2.3.1 Distribution Data for Endangered Species

The species list for this study was obtained from a Comprehensive Scientific Investigation of seven national nature reserves, namely Zhalong, Xianghai, Wuyuerhe, Minshui, Momog, Tumuji, and Korqin (Table S1). The selection of endangered species was guided by the following criteria: (1) inclusion of endangered species listed in the 2021 publication of the List of National Protected Key Wild Plants, (2) selection of species from the 2021 publication of the List of National List of Key Protected Wild Animals, and (3) reference to the IUCN Red List of Threatened Species (IUCN Red List). According to these criteria, a total of 37 species were chosen (Table S2). These species demonstrate high sensitivity to changes in the natural environment and serve as crucial indicators for the necessity of habitat protection (Hayward 2011).

Distribution records for endangered species were verified by combining previous searches (Comprehensive Scientific Investigation of seven national nature reserves, Global Biodiversity Information Facility at https://www.gbif.org/, China Bird Report from http://www.birdreport.cn/) and field surveys (two field surveys were conducted in July 2021 and January 2022). To avoid the adverse effects of spatial autocorrelation and pseudo‐replication of occurrence data on the model outcomes (Zhao, Xiao et al. 2022), data were sampled and filtered according to the following criteria. (1) Sampling was designed and conducted in accordance with the actual sampling environment in the Songnen Plain, with sampling points positioned at least 1 km apart. (2) The sampling outcomes were imported into ArcGIS 10.2, where the “buffer” tool was utilized to generate a 1 km buffer for each distribution point. Subsequently, these records were placed on a raster image of 30 m grid cell. (3) Species distribution points within 1 km were retained if there were fewer than three; if they were the same species, distribution points with overlapping spatial locations were removed. Finally, after removing height space autocorrelation and duplicate records, 29 species and 1574 occurrence records were retained for modeling (Table S2, Figure S1).

#### 2.3.2 Bioclimatic and Environmental Data

Our study considered 19 bioclimatic variables (Bio1‐19) and 8 environmental variables (Table 1, Table S3). Meteorological factors spanning the period from 1970 to 2000 were obtained from the WorldClim database (https://www.worldclim.org/) at a spatial resolution of 30 s. Road density and population density were obtained from the RESDC (http://www.resdc.cn). Soil texture was obtained from the Scientific Data Centre for Cold and Dry Zones (http://westdc.westgis.ac.cn/). The Normalized Difference Vegetation Index (NDVI) serves as an indicator of vegetation coverage and the spatial distribution of vegetation. It was derived by computing the average annual NDVI for China from 2018 to 2020, utilizing data supplied by the RESDC (http://www.resdc.cn).

**TABLE 1:** Environmental variables used in species distribution models.

Environmental factors	Indicator code	Data sources
Meteorological factors	Bio1‐19 Srad Wind Vapr	WorldClim (http://www.worldclim.org/version2)
Altitude	Alt	Geospatial Data Cloud, Computer Network Information Center, Chinese Academy of Sciences (http://www.gscloud.cn/search)
Normalized Difference Vegetation Index	NDVI	Resources and Environmental Science and Data Center of the Chinese Academy of Sciences (RESDC) (http://www.resdc.cn)
Road density	Road	Resources and Environmental Science and Data Center of the Chinese Academy of Sciences (RESDC) (http://www.resdc.cn)
Population density	Pode	Resources and Environmental Science and Data Center of the Chinese Academy of Sciences (RESDC) (http://www.resdc.cn)
Soil texture	Soil	Scientific Data Centre for Cold and Dry Zones (http://westdc.westgis.ac.cn/)

To avoid edge at the boundary of the Songnen Plain in the spatial analysis, the data extent was cropped by extending the Songnen Plain boundary for 20 km. The data were also unified into the CGCS2000 coordinate system prior to processing, ensuring that the coordinates and pixel dimensions of the raster data were uniform by interpolating the pixel dimensions into a resolution of 30 m × 30 m using Kriging (Hughes 2017). To reduce the interference of each environmental variable in the relevant data source (Sillero 2011), correlation coefficients were calculated using SPSS 24.0 software. Variables were removed based on Pearson's coefficient |*r*| > 0.8, retaining those factors that contributed most to the predicted probability (Sheppard and Gonzalez‐Andujar 2013). A total of 13 environmental variables were retained (Table S4).

#### 2.3.3 Model Set‐Up and Validation

The MaxEnt model is a machine learning tool used in the construction of SDMs (Phillips, Anderson, and Schapire 2006; Phillips et al. 2017). Previous studies have shown that MaxEnt performs well in using existing data to predict biological habitat suitability and species distribution (Pearson et al. 2007; Ejigu and Tassie 2020; Zhao, Xiao et al. 2022). The biological species data and corresponding environmental data used in this study were set as follows: (1) 75% of the data were randomly selected as the training group and 25% as the testing group; (2) the regularization multiplier (RM) was set to 1.1; (3) the mean of four replicate samples from the cross‐validation type of the model run was used for further analysis; (4) linear, quadratic product and correlation values; and (5) other default model parameters were left unchanged (Table S5).

**FIGURE 3:**
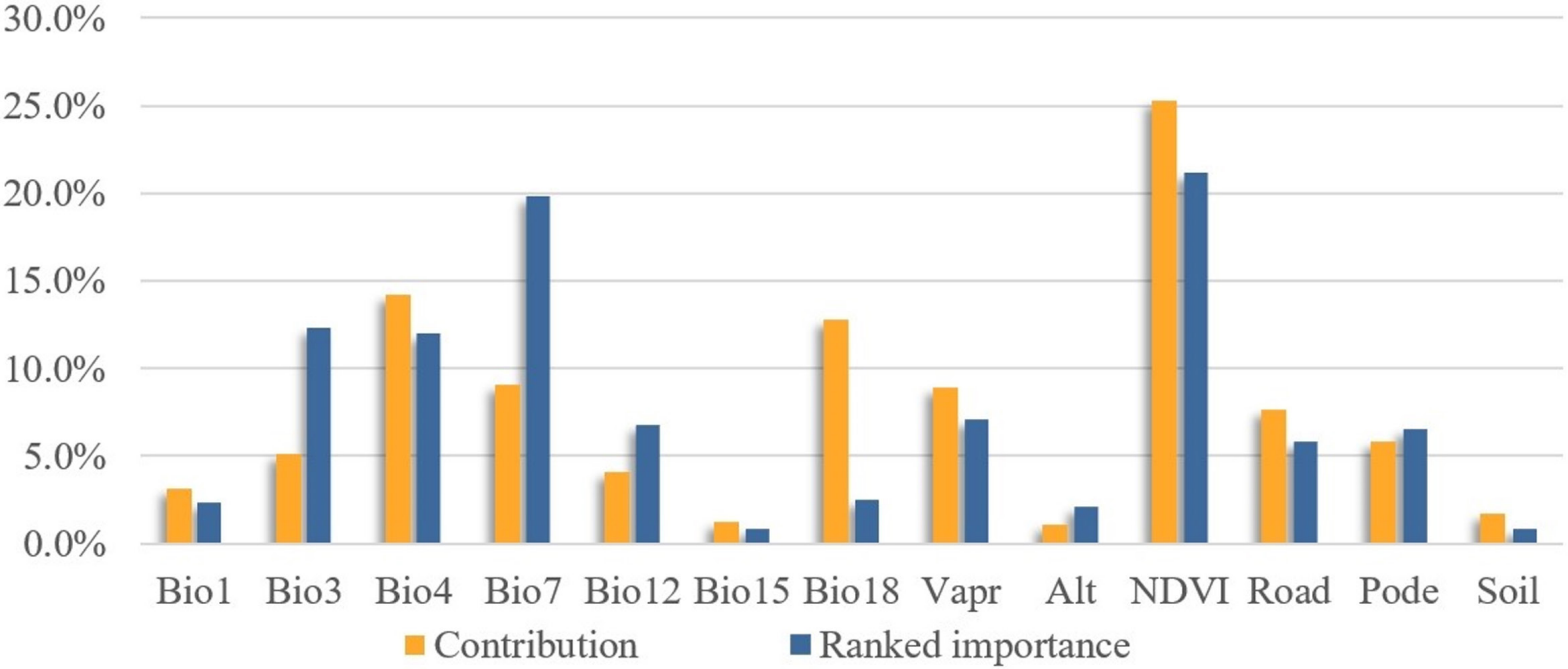
Influence of percentage contribution and permutation importance provided by environmental variables.

The Jackknife method was used to test the statistical significance of environmental variables and to reduce the influence of similar environmental variables on predicted results. Under complex species environmental conditions, the predictions are unlikely to be consistent with the actual distribution of species. Therefore, the reliability of species models is assessed by using the area under the curve (AUC) from receiver operating characteristic curve (ROC) analysis (Phillips and Dudı'k 2008). AUC values are used to reflect AUC values in the range [0, 1] (Liu et al. 2013), with larger values generally considered to indicate higher accuracy of model predictions (Luo et al. 2021). 0.75 < AUC < 0.85 is considered an acceptable prediction, and 0.85 < AUC < 1 indicates high accuracy (Swets 1988; Luo et al. 2021). In this study, predictions of potential distribution models were made for 29 species within the study area. The average training and test AUCs obtained for the four simulation iterations were 0.891 and 0.801, respectively, suggesting a satisfactory fit of the data to the model. Based on the contribution of the environmental variables to the maximum entropy model, eight variables stand out as the primary factors influencing species distribution. These variables provided a contribution of 88.8% and ranked importance of 87.2%. NDVI had the highest contribution (25.3%) and highest ranked importance (21.2%), followed by standard deviation of seasonal variation in temperature (Bio4) with a contribution of 14.2% and ranked importance of 12.0%, and the least influential was elevation (Alt) with a contribution of 1.1% and ranked importance of 2.1%, respectively (Figure 3). The output layer of the MaxEnt model was processed by using ArcGIS 10.2 to obtain a species diversity distribution map. Normalize the distribution hotspots of 29 species diversity and then add them with equal weights to obtain the predicted values of endangered species richness in the Songnen Plain area (Figures S2 and S3).

### 2.4 InVEST Model Prediction

In InVEST model prediction, the biodiversity module uses strengths and weaknesses in habitat quality to describe changes in the elasticity, resilience, breadth, and depth of biodiversity. Its calculation of habitat quality and habitat scarcity can be used to reflect biodiversity (Terrado et al. 2016; Sallustio et al. 2017). The land‐use/land‐cover (LULC) data for 2020 from China multi‐period land‐use remote sensing monitoring dataset (http://www.resdc.cn/DOI 10.12078/2018070201) were selected for analysis. In the InVEST model, habitat quality based on land‐use data was calculated as follows:

Habitatindex represents the habitat quality index:

$$Q_{\mathrm{xj}}=H_{J}\left(1-\frac{D_{\mathrm{xj}}^{z}}{D_{\mathrm{xj}}^{z}+k^{z}}\right)$$

where $Q_{\mathrm{xj}}$ denotes the habitat quality of raster *x* in land use and land use *j*; $H_{J}$ denotes the habitat suitability of land use *j*, ranging from 0 to 1; *z* is the scale constant; *k* is the half‐saturation constant, and when *k* = 0.5, the *k* value equals the *D* value; and $D_{\mathrm{xj}}$ is the habitat stress level of raster *x* in landscape type *j*.

$$D_{\mathrm{xj}}=\sum_{r=1}^{R}\sum_{y=1}^{Y_{r}}\left(\frac{W_{r}}{\sum_{r=1}^{R}W_{r}}\right)r_{y}i_{\mathrm{rxy}}\beta_{x}S_{\mathrm{jr}}$$

where *r* refers to the stressor; *R* refers to the number of stressors; $Y_{r}$ refers to the number of rasters of the stressor; *W*_*r*_ refers to the weight of the stressor; *r*_*y*_ refers to the number of stressors corresponding to a single raster cell; $\beta_{x}$ refers to the accessibility level of raster cell *x*, taking values between 0 and 1; $S_{\mathrm{jr}}$ refers to the sensitivity of stressor *r* corresponding to land‐use type *j*, taking values between 0 and 1; $i_{\mathrm{rxy}}$ refers to the influence distance of the stressor value $r_{y}$ of raster cell *y* on raster cell *x*, which can be specifically divided into linear decline and exponential decline calculations with the equations.

$$i_{\mathrm{rxy}}=1-\left(\frac{d_{\mathrm{xy}}}{d_{r\max}}\right)\left(\text{linear}\right)$$

$$i_{\mathrm{rxy}}=\exp\left[-\left(\frac{2.99}{d_{r\max}}\right)d_{\mathrm{xy}}\right]\left(\text{exponential}\right)$$

where $d_{\mathrm{xy}}$ is the linear distance between raster cell *x* and raster cell *y*; $d_{r\max}$ is the maximum influence distance of stressor *r*.

With reference to existing studies (Nematollahi et al. 2020; Zhao and Wu 2022) and the list of protected species in the Songnen Plain, the six categories of cultivated land, towns, villages, roads, railways, and mines were defined as threat sources for habitats. In addition, with reference to the studies of Wu et al. (2015) and Liu et al. (2017), the farthest threat distance, weights, attenuation of threat factors, and the sensitivity of different habitat categories to different threat factors were set (Table 2, Table S6). The weighting and sensitivity setting tables were saved in CSV format. The cultivated land, towns, villages, highways, railways, and mining sites used for threat factors are extracted from the data of land use and field research. All threat factor data were clipped to a 30 m × 30 m raster format with a spatial allocation of 1 for threat factors and 0 for other areas (the default is the smallest outer rectangle of raster data that does not contain threat factors).

**TABLE 2:** The scope and weight of threat sources.

Threat factors	Longest distance	Weight	Attenuation method
Field	5	0.3	Linear
Towns	12	1.0	Index
Villages and towns	10	0.9	Index
Roads	10	0.8	Linear
Railways	8	0.7	Linear
Mining sites	9	0.8	Index

### 2.5 Analysis of Key Areas of Conservation

In the conservation gap analysis, the results of the MaxEnt model layer were first overlaid with 2019 LULC data and areas of unsuitable species habitat were delineated with the aid of a “raster calculator” in ArcGIS 10.2 to avoid over‐prediction. In order to obtain more accurate suitable areas for biodiversity hotspots results, certain unsuitable habitats in the LULC data were excluded, such as city areas, capital farmland, and the buffer zone of the road within 1 km (Cao, Carver, and Yang 2019). In subsequent analyses, the distribution of at‐risk species was reclassified using a 10% training presence threshold (i.e., setting a value for the 10% of the local area that did not include the lowest predicted value as the threshold). This is generally considered to be an effective and conservative method for distinguishing suitable from unsuitable areas, thereby identifying critical conservation zones (Nazeri et al. 2012; Kramer‐Schadt et al. 2013; Radosavljevic and Anderson 2014). The method was employed to indicate predicted presence and was further categorized into four potential habitat classes: “unsuitable” (below threshold, 0.25), “less suitable habitat” (0.25–0.55), “moderately suitable habitat” (0.55–0.90), and “highly suitable habitat” (> 0.90).

InVEST model results overlap with the MaxEnt model result, and the results were integrated and classified by using the “Raster Calculator” and “Reclassify” tools in ArcGIS 10.2. The comparison was made against the existing boundaries of PAs in the Songnen Plain. This aimed to delineate over PAs (i.e., unsuitable habitat areas within PAs) and priority areas for biodiversity conservation (i.e., highly suitable habitat areas within PAs).

Determining conservation priorities: (1) areas jointly identified in the model portfolio are the “Core zone”; (2) areas identified by MaxEnt only or InVEST only are classified as the second priority; and (3) areas that fall within the management of PAs but are not identified by the model are classified as the third priority, corresponding to “key priority” and “priority,” respectively. According to the results obtained from the combined model in comparison with the PA boundaries, three tiers of priority zones were delineated within the Songnen Plain: Priority inside PAs < Key priority inside PAs < Priority inside core zone; Priority outside PAs < Key priority outside PAs < Priority outside core zone.

## 3 Results

### 3.1 MaxEnt Prediction Results

**FIGURE 4:**
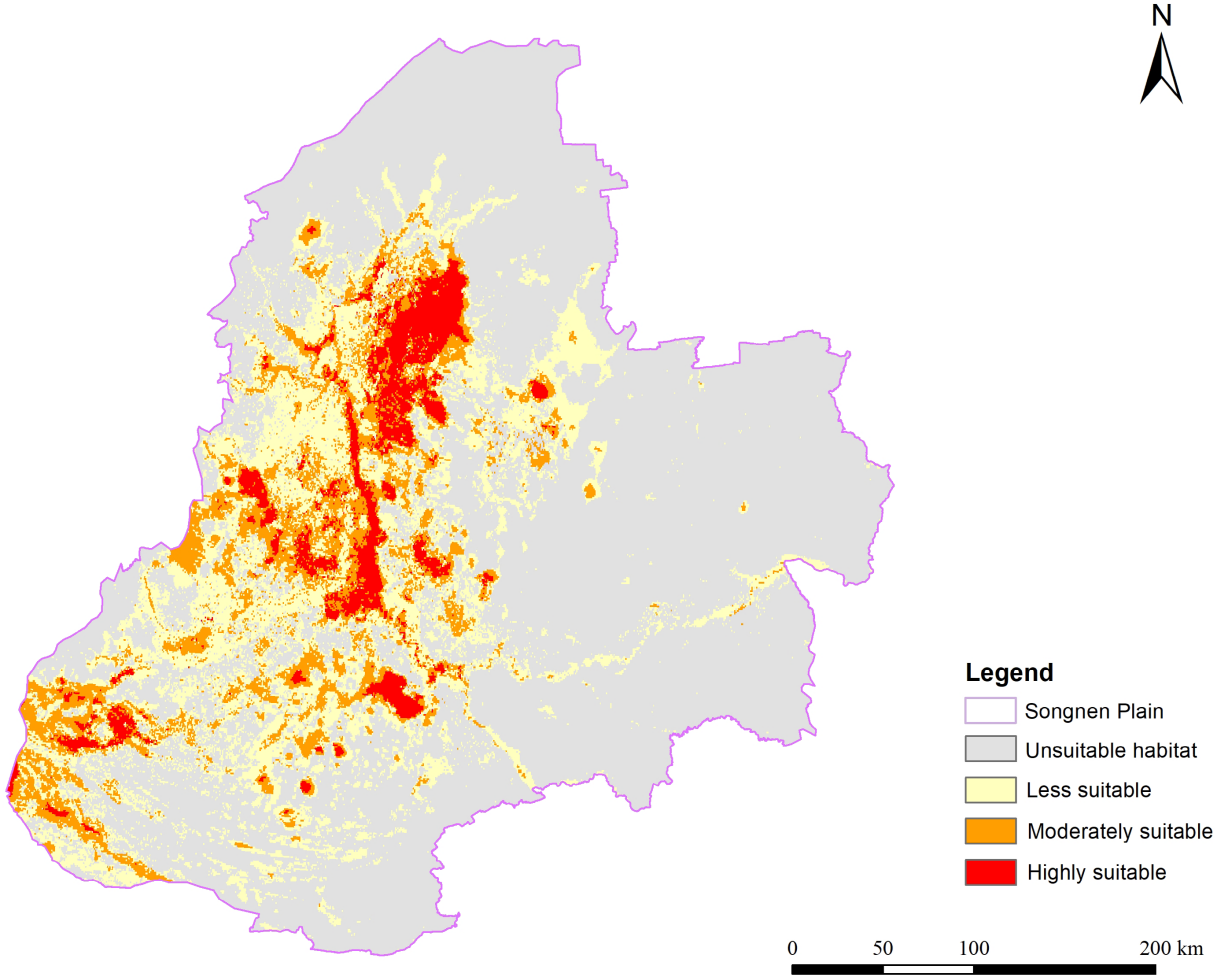
Location of four categories of suitable areas for biodiversity hotspots of endangered species.

The results showed that the suitable distribution areas of the endangered species accounted for 35.01% of the total area of the Songnen Plain. Species distribution hotspots were mainly situated in the central and southwestern parts of the Songnen Plain, with a total area of approximately 50,487.34 km^2^ (i.e., three categories of suitable areas; Figure 4). Concerning suitable habitats, the Songnen Plain was identified to contain a highly suitable habitat zone spanning 6390.65 km^2^, a moderately suitable habitat zone covering 15,587.02 km^2^, and a less suitable habitat zone extending for 28,509.66 km^2^. The highly suitable habitat zone and moderately suitable habitat zone were mainly situated in the central part of the Songnen Plain and the southwestern Songnen Plain near the Da Hinggan Range. The LULC in these areas was mainly wetlands and grasslands.

### 3.2 Integrated InVEST and MaxEnt Results

**FIGURE 5:**
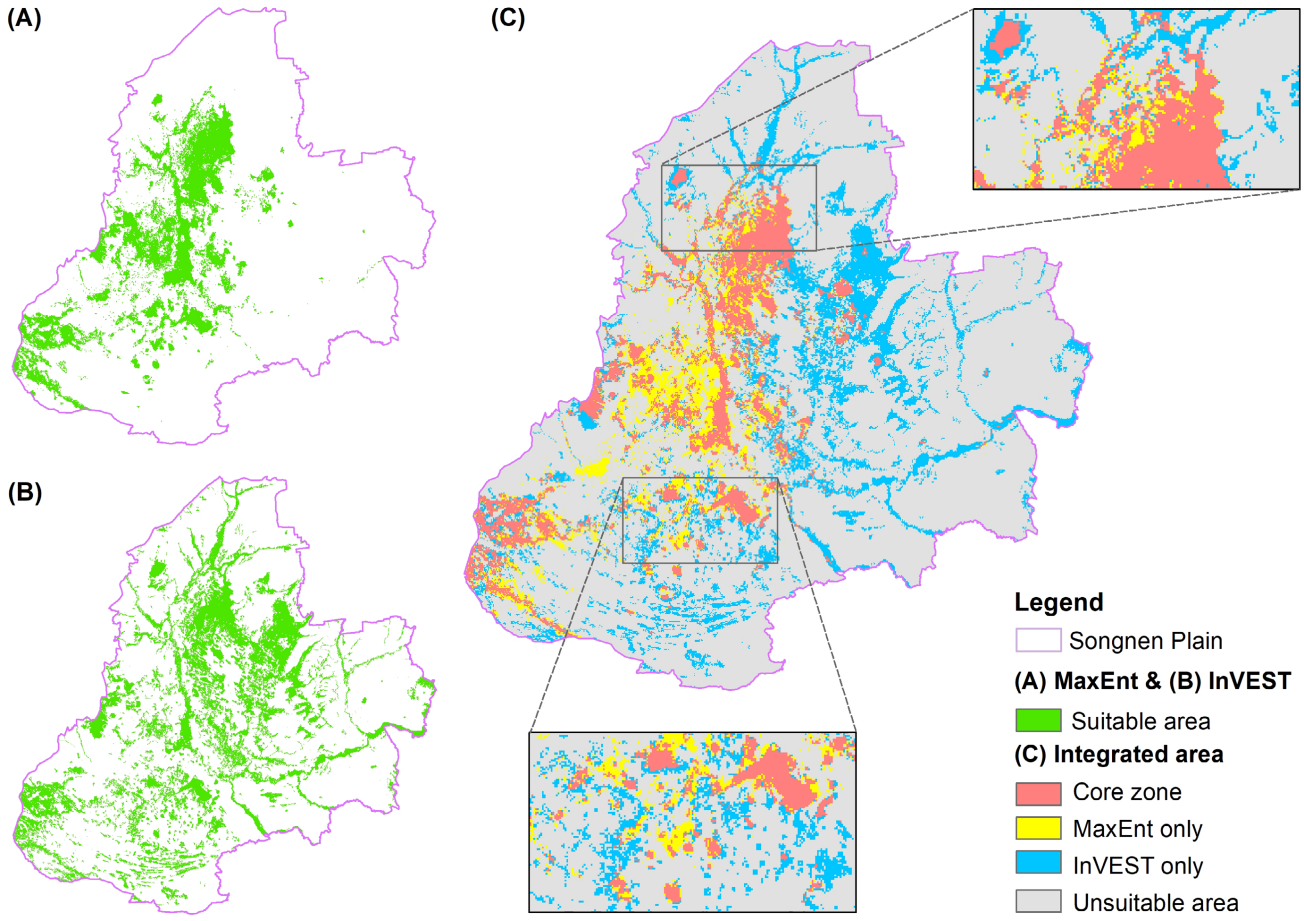
Overlapping of hotspot distribution maps obtained by the MaxEnt and InVEST models.

According to both the MaxEnt and InVEST models, habitat hotspots were concentrated in the central part of the Songnen Plain, followed by the southwestern region (Figure 5). A slight difference between the two model results was that the area of habitat hotspots in the southwestern grassland region was smaller in the InVEST model compared to the MaxEnt model. The overlay of the integrated modeling (MaxEnt‐InVEST) results indicated that the core zone area of conservation priority in the Songnen Plain was 14,764.14 km^2^, accounting for 10.24% of the total area of the Songnen Plain.

### 3.3 Identification of Conservation Priorities Areas

**FIGURE 6:**
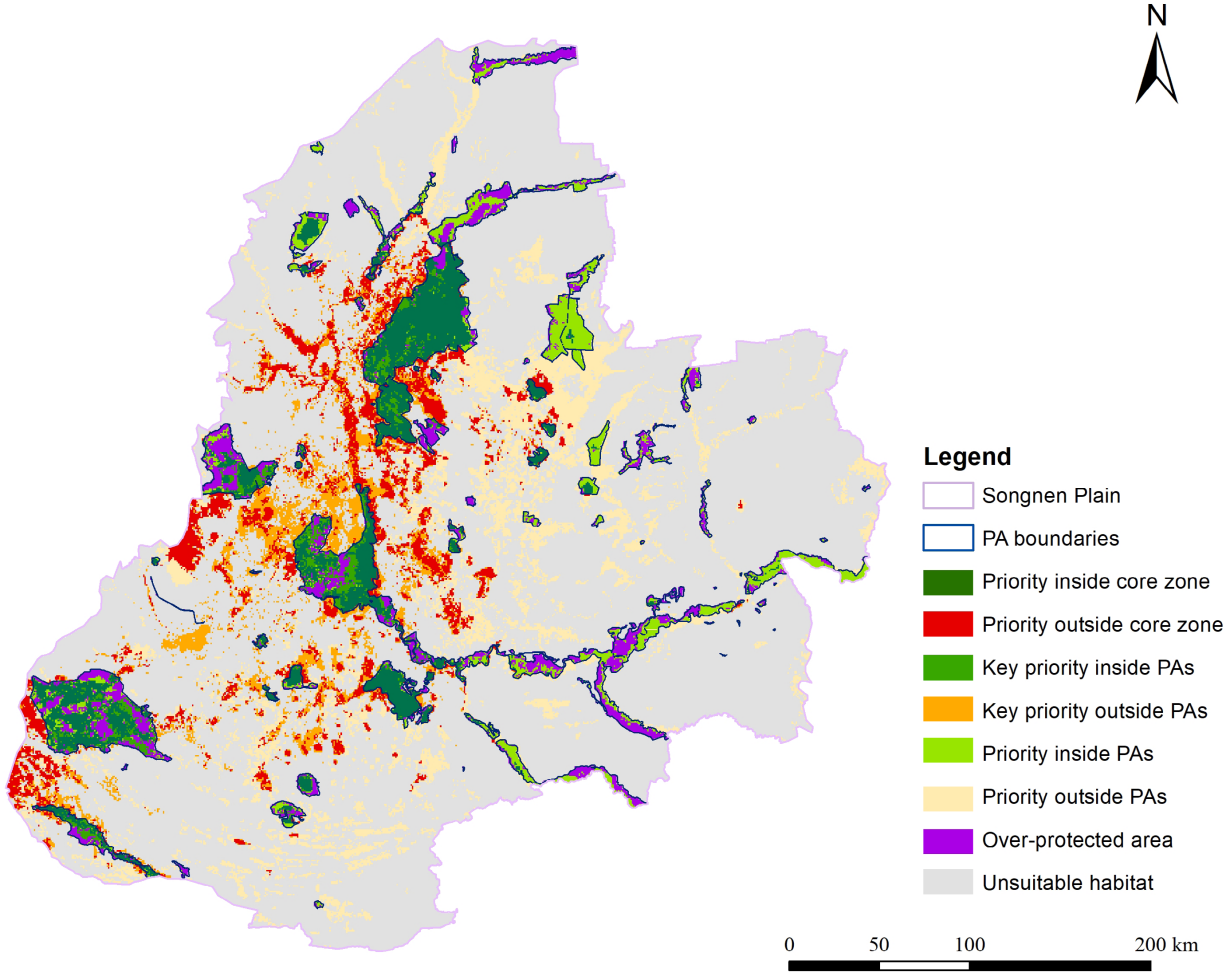
Overlapping hotspots with existing PAs to identify conservation priority areas.

The current boundaries of PAs were compared with the distribution of suitable hotspots derived from both models. The results showed that the simulated “Priority outside core zone” was mainly situated in the northeast, center, and southwest of the Songnen Plain, covering a total area of 7858.45 km^2^, constituting 53.22% of the species habitat area (5.45% of the total Songnen Plain area). The total area of “suitable habitat areas that do not belong to PAs” was 18,134.65 km^2^ (constituting 47.61% of the species' habitat area, 12.57% of the total area of the Songnen Plain), including “key priority outside PAs” with an area of 5517.53 km^2^ identified based on the MaxEnt model and “priority outside PAs” with an area of 12,617.12 km^2^ identified according to the InVEST model. The total area of existing PAs in the Songnen Plain was 16,617.90 km^2^, constituting 12.17% of the Songnen Plain area. Within the existing PAs, the total area protected by the reserve managers but not recognized as suitable habitat areas for the species was 4536.65 km^2^, accounting for 27.30% of the existing PAs in the Songnen Plain. The gap analysis highlighted that nearly half of the study area (47.61%) lacked legal protection (Figure 6).

## 4 Discussion

### 4.1 Reasons of Conservation Gaps in the Songnen Plain

Our results showed that the designated “priority outside core zone” encompassed 53.22% of the habitat area for species in the Songnen Plain and constituted 5.45% of the total land area of the Songnen Plain. This highlighted a serious conservation gap within the region. The total area of existing PAs in the Songnen Plain was 16,617.90 km^2^, constituting 12.17% of the area of the Songnen Plain, which was notably lower than the national average of 20% for nature reserves. We summarized the reasons for the conservation gaps as follows.

First, the lack of adequate information on protected species (including species, numbers, and distribution) makes it difficult to use this as a valid scientific basis for establishing separate PA boundaries. This ultimately leads to discrepancies in spatial location between planned PA boundaries and the actual distribution of biodiversity hotspots. The poor accuracy of early geographic information systems to some extent affected the boundary delineation of PAs. The decentralized management of PAs before the introduction of the national park system may also have led to the expedited deterioration of natural habitats within the Songnen Plain PAs and changes in the actual distribution patterns of endangered species (Lu et al. 2015; Xu et al. 2019). Crop cultivation and animal husbandry remained integral to the daily routines of the local inhabitants. In an effort to boost their income and support the growing rural and urban communities, local residents endeavored to enlarge agricultural areas and augment livestock numbers, thereby contributing to the economic development of the region (Zhao, Wang et al. 2022). In the past 50 years, the farmland and built‐up areas in the Songnen Plain have increased by 10% and 2.7%, respectively, while wetlands and grasslands have experienced a respective reduction of 7.8% and 16.3% (Wang et al. 2011). Changes in LULC increase the conservation gaps and pose greater challenges to the PA conservation objectives for endangered species in the Songnen Plain.

Second, there has been a lack of long‐term, effective, and sustainable conservation planning in PAs, particularly in areas where management funding is insufficient. Continuously investing funds and developing reasonable protection plans to ensure the effectiveness of PAs is far more difficult than merely establishing them (Catullo et al. 2008). While the majority of endangered species identified in our study were recognized as conservation targets during the initial establishment of the Songnen Plain PAs, the long‐term development strategy of the PAs frequently overlooks the comprehensive protection of these species and their habitats (Araújo et al. 2011; Alatawi, Gilbert, and Reader 2020). The master plans for many PAs were short‐term (5–10 years), and some PAs only developed a master plan after their establishment. As a result, their efficacy in conservation was limited, leading to conservation gaps.

### 4.2 Spatial Optimization of PAs in the Songnen Plain

Our results revealed significant conservation gaps in the Songnen Plain. The highest conservation priorities outside PAs were observed in the central part of the Songnen Plain, followed by the southwest. To reduce these conservation gaps and align with China's recent reform measures for the PAs system (which categorizes PAs into national parks, nature reserves, and natural parks), we propose adjusting and optimizing the existing PAs in the Songnen Plain as follows:

First, establish new PAs that balance economic development with ecosystem services. Our approach will help reduce conservation gaps and strengthen on‐site species conservation within PAs. (1) Integrate seven national nature reserves into national parks to enhance the protection of rare species and increase the inclusiveness of the PA network. (2) Based on the distribution and patch size of the “core zone,” identify five areas as potential sites for new nature reserves, covering approximately 230 km^2^ in total. Additionally, select 23 potential sites for new nature parks, covering roughly 1200 km^2^.

Second, adjustments should be made to the spatial boundaries and administrative levels of existing PAs. An analysis of the proportion of conservation priority areas within individual PAs revealed the following: (1) There were 58 priorities with an area proportion of more than 75%—42 maintained the status quo, 13 needed to be adjusted in level, and 3 needed to be adjusted in type. (2) There were 33 priorities with an area proportion between 25% and 75% 28 maintained the status quo, two needed to be adjusted in level, and three needed to be adjusted in type. (3) There were six priorities with an area proportion of less than 25%—one needed to be adjusted in level, one needed to be adjusted in type, and three were recommended for downgrading or withdrawal.

Third, develop reasonable management plans based on the type and level of PAs. We recommend properly controlling anthropogenic activities within or around PAs to minimize potential interference with species' habitats. Since there is still farmland, reservoirs, and even economic forests within the PA, stricter access permits or reward and punishment systems should be established for relevant management measures. For example, residents could be encouraged to report illegal activities, and severe penalties should be imposed for violations. For construction land, production land, and other LULC that cannot be conveniently included in the PAs, strict control should be exercised over lighting, noise, and other factors to avoid significant negative impacts on the biological rhythms and foraging and breeding behaviors of species.

### 4.3 Differences Between MaxEnt and InVEST Model

In the combined modeling framework of this study, the MaxEnt model demonstrated effective simulation of potential species distributions influenced by natural climate factors, and the InVEST model performed well in simulating habitat quality for ecosystem support services by using current land‐use data. MaxEnt models are designed to assess species presence in potential ranges where large‐scale field surveys are challenging or impractical. To enhance the accuracy of the MaxEnt model in predicting species distribution in plain areas, we selected a wide range of environmental variables and conducted correlation analyses. This approach allowed us to independently predict the distribution of each species by reducing potential biases and ensuring that the environmental factors most relevant to species habitat were included in the model. The output generates a high probability of species distribution hotspots, which can be considered biodiversity hotspots (Phillips, Anderson, and Schapire 2006; Han et al. 2019). However, when employed for conservation gap analysis, the MaxEnt model often overlook actual land‐use data, leading to predictions that may overestimate outcomes.

To correct for the over‐predictions that result in exaggerated data on actual conservation gaps, integrating models with environmental indicators, such as habitat quality, is highly beneficial for effective conservation planning (Sallustio et al. 2017; Gong et al. 2019). Based on this, the present study utilized the InVEST model, incorporating various land‐use sensitivities and threats to map habitat quality (Masiero et al. 2019). This integration aimed to address the over‐predictions produced by the MaxEnt model. The combined approach met the requirements of PAs for conservation objectives and assessed potential values associated with ecosystem services (Xu et al. 2017; Masiero et al. 2019).

In accordance with the results of this study, the fit between the InVEST and MaxEnt models was 72.11%. This gap reflects the fact that the uncertainty in setting the parameter settings for different land‐use threats when using the InVEST model, which hinders the fit of the model's predictions to the actual situation compared to the species distribution data observed in the field surveys. Therefore, the integration of the two models in this study's framework was employed to mutually enhance their strengths, leading to more precise predictions of biodiversity hotspots. Compared to other studies, the identification of priority PAs in the Songnen Plain using our combined model reveals notable differences in overlapping areas. For instance, the proportion of overlap is significantly smaller than that observed in the Xishuangbanna tropical area (Huang et al. 2020). This discrepancy may be attributed to the high intensity of human influence on the Songnen Plain (Huang, Qian, and Cao 2022). This underscores the complex challenges posed by conservation planning in heavily urbanized or human‐influenced regions.

### 4.4 Strengths and Limitations of the Conservation Framework

Our research framework for evaluating regional PA network can be applied to numerous similar areas where PAs are insufficient to protect habitats for biodiversity. We utilized the combined model (MaxEnt‐InVEST) to integrate its biodiversity and habitat quality hotspots, fully considering the potential distribution of rare species under the synergistic effects of natural and human interference conditions and improving the accuracy of identifying rare species diversity hotspots and protection gaps. Furthermore, the results of the combined model also facilitate the exploration of the potential impact of biodiversity hotspots and the balancing of LULC needs with ecosystem services.

Admittedly, our study has some limitations. First, only 27 environment variables were used in this study, mainly related to temperature, precipitation, and geography. Additional environmental variables, such as water quality classes, future climate data, global climate models, and anthropogenic disturbances, could also be considered (Han et al. 2019). Second, in predicting biodiversity hotspots, our study only focused on rare and protected flora and fauna covered in the Comprehensive Scientific Investigation of the seven national nature reserves. A broader range of threatened wildlife, such as reptiles and amphibians, could be included in future assessments to optimize biodiversity conservation (Hughes 2017). Third, while the MaxEnt model is effective in predicting species distributions, it carries uncertainties, especially in its assumptions regarding data (Elith et al. 2006; Yackulic et al. 2013). The model assumes that data comes solely from random sampling and that environmental covariates remain constant. If these conditions are not met, the predictions could be skewed. Thus, for conservation gap analysis in PAs, using field‐collected data to build the model is preferable to relying on potentially outdated data stored in museums or herbaria. Moreover, the InVEST model also has data limitations, such as the need for more precise information. In this study, more accurate data were unavailable, with land‐use type classifications and parameter sensitivity largely based on expert knowledge of the Songnen Plain (Gong et al. 2019). Future model integration should address land‐use changes and their powerful effects, providing more precise data for conservation assessments (Sallustio et al. 2017; Xu et al. 2017). Finally, a more detailed analysis is needed to evaluate the biodiversity hotspots identified through model evaluation for spatial optimization of PAs. While this study proposed a spatial adjustment and optimization plan for PAs in the Songnen Plain based on current PA classification and policies, as well as the proportion of priority areas within each PA, we did not consider the economic costs of constructing PAs. Therefore, integrating a Systematic Conservation Planning framework with Marxan or Zonation models could help optimize and adjust the PA network in a cost‐effective manner.

## 5 Conclusion

In our study, the combined MaxEnt‐InVEST model was used to identify key areas for biodiversity conservation in the Songnen Plain and to assess the effectiveness of the current PAs. The results indicated that endangered species require significant attention, and the spatial management scope of nature reserves needs to be adjusted based on the distribution of these protected species. The method also demonstrated that species distribution data are effective for evaluating existing PAs and informs the development of more precise spatial planning for the PA network. According to the analysis, the total area covered by species hotspots and habitat hotspots was 14,764.14 km^2^, accounting for 10.24% of the Songnen Plain. Species hotspots alone (from MaxEnt) covered 7213.53 km^2^, or 5.01% of the region, while habitat hotspots alone (from InVEST) covered 16,113.49 km^2^, or 11.17% of the Songnen Plain. For the endangered species analyzed, the existing PAs in the Songnen Plain did not provide sufficient protection.

The research results also revealed the conservation gap in the Songnen Plain and the necessity of optimizing the spatial pattern of nature reserves, which is a direction that needs further attention. The combined MaxEnt‐InVEST model developed in this study can respect the current situation of land use in the region, identify and map key conservation areas, and enhance the precision of evaluating biodiversity hotspots based on actual species distribution data. This method can serve as a standard component of biodiversity assessment and conservation methods and can also provide a scientific basis for optimizing the spatial pattern of nature reserves. Therefore, it can assist the Songnen Plain in building a PA system centered on national park.

## Author Contributions

**Qiaoyun Sun:** conceptualization (equal), funding acquisition (equal), investigation (equal), methodology (equal), validation (equal), writing – original draft (equal), writing – review and editing (lead). **Jianqi Yu:** conceptualization (equal), validation (equal), writing – original draft (equal), writing – review and editing (equal). **Yingran Zeng:** investigation (equal), methodology (equal), writing – original draft (supporting). **Yifang Gai:** investigation (equal), visualization (equal). **Jia Wang:** visualization (equal), writing – original draft (equal). **Yujun Zhang:** conceptualization (equal), funding acquisition (equal), supervision (equal), writing – review and editing (equal).

## Conflicts of Interest

The authors declare no conflicts of interest.

## Supporting information


Data S1.

## Data Availability

The data that supports the findings of this study are available in the Supporting Information of this article.
