# Supplementary material for: Mapping Biodiversity Conservation Priorities for Protected Areas for Spatial Optimization: A Case Study in the Songnen Plain, China
Source: Ecol Evol. 2024 Nov 5;14(11):e70516. doi: 10.1002/ece3.70516 (PMC11537694; doi:10.1002/ece3.70516)
Supplement: Supplementary file 1 — Data S1. [file ECE3-14-e70516-s001.docx]

Supplementary Material

**Table S1** Details of 7 national nature reserves in the Songnen Plain

| No. | Name | Area (km^2^) | Establi-shment year | Year of Scientific Investiga-tion | Species | | | | | | | |
| --- | --- | --- | --- | --- | --- | --- | --- | --- | --- | --- | --- | --- |
|  |  |  |  |  | Mo-sses | Ferns | Seed plants | Fish | Amphibians | Reptiles | Birds | Mammals |
| 1 | Zhalong | 2185.94 | 1979 | 2017 | 8 | 2 | 466 | 46 | 6 | 2 | 260 | 21 |
| 2 | Xianghai | 1054.68 | 1981 | 2016 | 11 | 3 | 623 | 29 | 5 | 8 | 293 | 37 |
| 3 | Wuyuerhe | 553.89 | 1990 | 2011 | 20 | 3 | 478 | 63 | 6 | 3 | 265 | 25 |
| 4 | Minshui | 305.57 | 2003 | 2011 | 6 | 4 | 524 | 28 | 6 | 6 | 229 | 32 |
| 5 | Momog | 1440.11 | 1981 | 2019 | / | 4 | 501 | 52 | 6 | 8 | 305 | 31 |
| 6 | Tumuji | 948.25 | 1996 | 2018 | / | 6 | 484 | 20 | 6 | 8 | 260 | 23 |
| 7 | Korqin | 1269.86 | 1985 | 2019 | / | 2 | 495 | / | 7 | 10 | 225 | 43 |
|  | Total |  |  |  | 20 | 7 | 1040 | 85 | 10 | 11 | 349 | 58 |

**Note:** The total area of existing PAs in the Songnen Plain is 16617.90 km^2^, accounting for 12.17% of the Songnen Plain area. According to surveys and statistics, there are 139 PAs in the Songnen Plain, divided into 4 levels (national, provincial, prefecture, and county) and 8 categories (natural reserves, national park of China, water conservancy scenic area, geological park, forest park, wetland park, desert park, and aquatic species resource protection area). The protected species in national nature reserves are relatively representative, so we choose national nature reserves as the source of species data list.

**Table S2** Set of 37 endangered wild animals targeted in this study

| Species | Protection level | IUCN | Residence type | Records | Records for model |
| --- | --- | --- | --- | --- | --- |
| *Lutra lutra* | Ⅱ | NT | / | 5 | / |
| *Anser albifrons* | Ⅱ | LC | T | 32 | 31 |
| *Mergus squamatus* | Ⅰ | EN | S | 28 | 28 |
| *Otis tarda* | Ⅰ | VU | S | 64 | 58 |
| *Grus leucogeranus* | Ⅰ | CR | T | 44 | 43 |
| *Grus vipio* | Ⅰ | VU | S | 96 | 89 |
| *Grus japonensis* | Ⅰ | VU | S | 148 | 142 |
| *Grus monacha* | Ⅰ | VU | T | 29 | 28 |
| *Numenius minutus* | Ⅱ | LC | T | 36 | 33 |
| *Ciconia nigra* | Ⅰ | LC | S | 29 | 28 |
| *Ciconia boyciana* | Ⅰ | EN | S | 89 | 83 |
| *Platalea leucorodia* | Ⅱ | LC | S | 120 | 111 |
| *Platalea minor* | Ⅰ | EN | S | 34 | 31 |
| *Egretta eulophotes* | Ⅰ | VU | T | 5 | / |
| *Pandion haliaetus* | Ⅱ | LC | S | 36 | 29 |
| *Aegypius monachus* | Ⅰ | NT | S | 95 | 83 |
| *Clanga clanga* | Ⅰ | VU | W | 29 | 27 |
| *Nisaetus nipalensis* | Ⅱ | NT | T | 11 | / |
| *Aquila nipalensis* | Ⅰ | EN | S | 31 | 29 |
| *Aquila heliaca* | Ⅰ | VU | S | 2 | / |
| *Aquila chrysaetos* | Ⅰ | LC | S | 21 | 20 |
| *Circus aeruginosus* | Ⅱ | LC | S | 40 | 37 |
| *Circus spilonotus* | Ⅱ | LC | S | 102 | 97 |
| *Circus cyaneus* | Ⅱ | LC | S | 88 | 81 |
| *Circus melanoleucos* | Ⅱ | LC | S | 91 | 85 |
| *Haliaeetus leucoryphus* | Ⅰ | EN | S | 2 | / |
| *Haliaeetus albicilla* | Ⅰ | LC | S | 9 | / |
| *Butastur indicus* | Ⅱ | LC | S | 29 | 24 |
| *Buteo lagopus* | Ⅱ | LC | W | 71 | 69 |
| *Buteo hemilasius* | Ⅱ | LC | S | 36 | 36 |
| *Buteo buteo* | Ⅱ | LC | S | 40 | 38 |
| *Strix uralensis* | Ⅱ | LC | R | 27 | 26 |
| *Strix nebulosa* | Ⅱ | LC | R | 13 | / |
| *Glaucidium passerinum* | Ⅱ | LC | R | 6 | / |
| *Falco tinnunculus* | Ⅱ | LC | S | 159 | 132 |
| *Falco rusticolus* | Ⅰ | LC | W | 25 | 24 |
| *Falco peregrinus* | Ⅱ | LC | T | 37 | 32 |
| Total |  |  |  | 1759 | 1574 |

**Note:** Protection: species with higher level (Ⅰ) indicates more endangered. Evaluation of the International Union for Conservation of Nature (IUCN) Red-list of criteria (NE: not evaluated; DD: data deficient; LC: least concern; NT: near threatened; VU: vulnerable; EN: endangered; CR: critically endangered; EW: extinct in the wild). Residence type: the migration behavior of birds in the Songnen Plain (S: summer visitor; W: winter visitor; R: resident; T: transient)


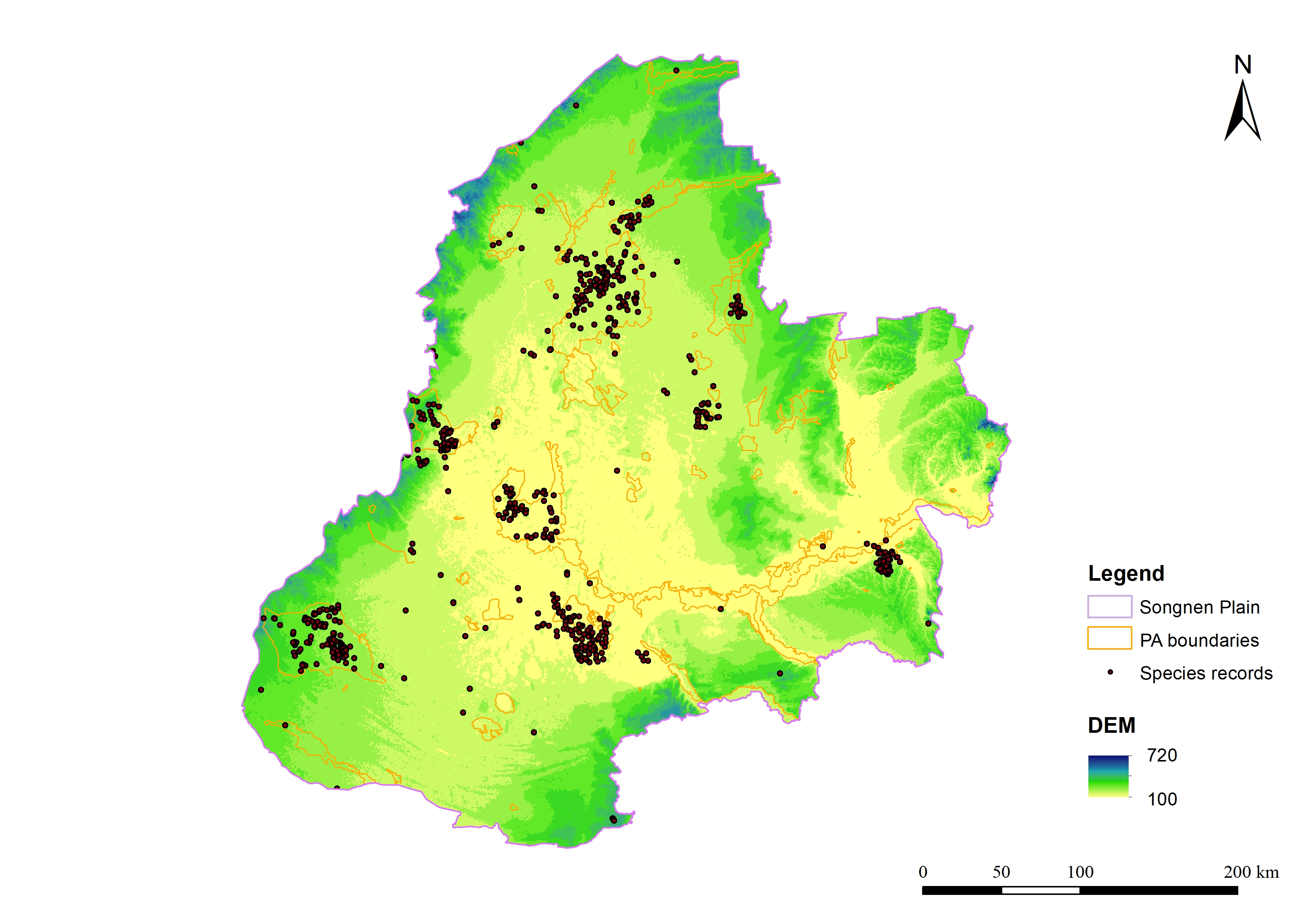


**Figure S1** Spatial distribution of 1759 occurrence records (29 rare and endangered species, include 1574 records for model) of endangered wild animals used for modeling.

**Table S3** Environmental variables used in modeling

| Variable | Description |
| --- | --- |
| **Bio1** | Annual mean temperature |
| Bio2 | Annual mean diurnal range |
| **Bio3** | Isothermality |
| **Bio4** | Temperature seasonality |
| Bio5 | Max temperature of warmest month |
| Bio6 | Min temperature of coldest month |
| **Bio7** | Annual temperature range |
| Bio8 | Mean temperature of wettest quarter |
| Bio9 | Mean temperature of driest quarter |
| Bio10 | Mean temperature of warmest quarter |
| Bio11 | Mean temperature of coldest quarter |
| **Bio12** | Annual precipitation |
| Bio13 | Precipitation in wettest month |
| Bio14 | Precipitation in driest month |
| **Bio15** | Precipitation seasonality (CV) |
| Bio16 | Precipitation of wettest quarter |
| Bio17 | Precipitation in driest quarter |
| **Bio18** | Precipitation in warmest quarter |
| Bio19 | Precipitation in coldest quarter |
| Srad | Solar radiation |
| Wind | Wind speed |
| **Vapr** | Vapor pressure |
| **Alt** | Altitude |
| **NDVI** | Normalized Difference Vegetation Index |
| **Road** | Road density |
| **Pode** | Population density |
| **Soil** | Soil texture |

**Note:** Unbolded variables have been removed from the model.

**Table S4** Pearson correlation coefficients for the 27 environmental variables listed in Table S3

| Variables | Bio1 | Bio2 | Bio3 | Bio4 | Bio5 | Bio6 | Bio7 | Bio8 | Bio9 | Bio  10 | Bio  11 | Bio  12 | Bio  13 | Bio  14 | Bio  15 | Bio  16 | Bio  17 | Bio  18 | Bio  19 | Srad | Wind | Vapr | Alt | NDVI | Road | Pode | Soil |
| --- | --- | --- | --- | --- | --- | --- | --- | --- | --- | --- | --- | --- | --- | --- | --- | --- | --- | --- | --- | --- | --- | --- | --- | --- | --- | --- | --- |
| **Bio1** | 1 |  |  |  |  |  |  |  |  |  |  |  |  |  |  |  |  |  |  |  |  |  |  |  |  |  |  |
| Bio2 | 0.87 | 1 |  |  |  |  |  |  |  |  |  |  |  |  |  |  |  |  |  |  |  |  |  |  |  |  |  |
| **Bio3** | 0.54 | 0.78 | 1 |  |  |  |  |  |  |  |  |  |  |  |  |  |  |  |  |  |  |  |  |  |  |  |  |
| **Bio4** | -0.16 | -0.39 | -0.77 | 1 |  |  |  |  |  |  |  |  |  |  |  |  |  |  |  |  |  |  |  |  |  |  |  |
| Bio5 | 0.88 | 0.25 | 0.50 | -0.68 | 1 |  |  |  |  |  |  |  |  |  |  |  |  |  |  |  |  |  |  |  |  |  |  |
| Bio6 | 0.94 | 0.20 | 0.62 | -0.94 | 0.83 | 1 |  |  |  |  |  |  |  |  |  |  |  |  |  |  |  |  |  |  |  |  |  |
| **Bio7** | -0.75 | -0.14 | -0.60 | 0.67 | -0.63 | -0.76 | 1 |  |  |  |  |  |  |  |  |  |  |  |  |  |  |  |  |  |  |  |  |
| Bio8 | 0.92 | -0.04 | 0.27 | -0.62 | 0.90 | 0.79 | -0.64 | 1 |  |  |  |  |  |  |  |  |  |  |  |  |  |  |  |  |  |  |  |
| Bio9 | 0.91 | 0.38 | 0.71 | -0.92 | 0.81 | 0.92 | -0.85 | 0.75 | 1 |  |  |  |  |  |  |  |  |  |  |  |  |  |  |  |  |  |  |
| Bio10 | 0.93 | -0.04 | 0.27 | -0.62 | 0.90 | 0.79 | -0.64 | 0.96 | 0.75 | 1 |  |  |  |  |  |  |  |  |  |  |  |  |  |  |  |  |  |
| Bio11 | 0.97 | 0.28 | 0.67 | -0.96 | 0.84 | 0.98 | -0.92 | 0.82 | 0.95 | 0.81 | 1 |  |  |  |  |  |  |  |  |  |  |  |  |  |  |  |  |
| **Bio12** | -0.37 | -0.32 | -0.49 | 0.48 | -0.53 | -0.55 | 0.48 | -0.30 | -0.48 | -0.29 | -0.47 | 1 |  |  |  |  |  |  |  |  |  |  |  |  |  |  |  |
| Bio13 | -0.19 | -0.31 | -0.39 | 0.30 | -0.41 | -0.36 | 0.28 | -0.14 | -0.30 | -0.14 | -0.28 | 0.89 | 1 |  |  |  |  |  |  |  |  |  |  |  |  |  |  |
| Bio14 | -0.40 | -0.29 | -0.46 | 0.47 | -0.49 | -0.53 | 0.48 | -0.32 | -0.48 | -0.31 | -0.47 | 0.87 | 0.69 | 1 |  |  |  |  |  |  |  |  |  |  |  |  |  |
| **Bio15** | 0.30 | 0.40 | 0.56 | -0.50 | 0.35 | 0.49 | -0.50 | 0.15 | 0.45 | 0.15 | 0.43 | -0.73 | -0.65 | -0.77 | 1 |  |  |  |  |  |  |  |  |  |  |  |  |
| Bio16 | -0.36 | -0.25 | -0.40 | 0.41 | -0.55 | -0.51 | 0.41 | -0.32 | -0.44 | -0.31 | -0.43 | 0.98 | 0.93 | 0.83 | -0.76 | 1 |  |  |  |  |  |  |  |  |  |  |  |
| Bio17 | -0.42 | -0.33 | -0.52 | 0.52 | -0.53 | -0.58 | 0.53 | -0.32 | -0.52 | -0.32 | -0.51 | 0.94 | 0.77 | 0.93 | -0.89 | 0.90 | 1 |  |  |  |  |  |  |  |  |  |  |
| **Bio18** | -0.36 | -0.25 | -0.40 | 0.41 | -0.55 | -0.51 | 0.41 | -0.32 | -0.44 | -0.32 | -0.43 | 0.78 | 0.79 | 0.77 | -0.76 | 0.83 | 0.79 | 1 |  |  |  |  |  |  |  |  |  |
| Bio19 | -0.42 | -0.33 | -0.52 | 0.52 | -0.53 | -0.58 | 0.53 | -0.33 | -0.52 | -0.33 | -0.51 | 0.94 | 0.77 | 0.93 | -0.89 | 0.90 | 0.98 | 0.92 | 1 |  |  |  |  |  |  |  |  |
| Srad | 0.50 | 0.29 | 0.51 | -0.58 | 0.62 | 0.64 | -0.57 | 0.41 | 0.60 | 0.41 | 0.58 | -0.94 | -0.76 | -0.83 | 0.82 | -0.92 | -0.91 | -0.92 | -0.90 | 1 |  |  |  |  |  |  |  |
| Wind | 0.81 | 0.72 | 0.63 | 0.05 | 0.02 | 0.53 | 0.88 | 0.23 | 0.46 | 0.21 | 0.47 | 0.03 | 0.21 | 0.82 | -0.42 | 0.75 | 0.01 | 0.74 | 0 | -0.12 | 1 |  |  |  |  |  |  |
| **Vapr** | 0.33 | -0.53 | -0.45 | 0.07 | 0.22 | 0.06 | 0.02 | 0.53 | 0.07 | 0.53 | 0.14 | 0.45 | 0.51 | 0.33 | -0.52 | 0.38 | 0.44 | 0.38 | 0.41 | -0.31 | 0.33 | 1 |  |  |  |  |  |
| **Alt** | -0.11 | 0.41 | 0.35 | -0.11 | -0.08 | 0 | -0.04 | -0.26 | 0.05 | -0.25 | -0.01 | -0.23 | -0.28 | -0.20 | 0.25 | -0.21 | -0.25 | -0.20 | -0.25 | 0.21 | 0.46 | -0.45 | 1 |  |  |  |  |
| **NDVI** | -0.17 | -0.09 | -0.18 | 0.22 | -0.03 | -0.16 | 0.21 | -0.07 | -0.16 | -0.08 | -0.20 | -0.15 | -0.17 | -0.12 | 0.05 | -0.19 | -0.12 | -0.18 | -0.12 | 0.11 | 0.14 | 0 | 0.03 | 1 |  |  |  |
| **Road** | -0.03 | 0 | 0 | -0.01 | 0 | 0 | -0.01 | -0.04 | 0 | -0.05 | 0 | -0.07 | -0.11 | -0.04 | 0.04 | -0.09 | -0.05 | -0.09 | -0.04 | 0.07 | 0.12 | -0.08 | 0.03 | 0 | 1 |  |  |
| **Pode** | 0.13 | 0.05 | 0.11 | -0.15 | 0.22 | 0.18 | -0.14 | 0.13 | 0.16 | 0.13 | 0.16 | -0.21 | -0.23 | -0.15 | 0.14 | -0.23 | -0.18 | -0.23 | -0.19 | 0.24 | 0 | -0.08 | -0.05 | 0.02 | 0.18 | 1 |  |
| **Soil** | -0.29 | 0 | -0.12 | 0.24 | -0.23 | -0.26 | 0.24 | -0.27 | -0.24 | -0.27 | -0.26 | 0.02 | -0.04 | 0.05 | -0.02 | 0.01 | 0.05 | 0.01 | 0.06 | -0.08 | -0.03 | -0.14 | 0.08 | 0.11 | 0.02 | -0.02 | 1 |

**Table S5** Modeling parameters for 29 species

| Species | linear/quadratic/product | categorical | threshold | hinge |
| --- | --- | --- | --- | --- |
| Anser albifrons | 1.000 | 0.530 | 1.920 | 0.500 |
| Aegypius monachus | 0.269 | 0.250 | 1.710 | 0.500 |
| Aquila chrysaetos | 1.000 | 0.560 | 1.940 | 0.500 |
| Aquila nipalensis | 1.000 | 0.575 | 1.950 | 0.500 |
| Butastur indicus | 1.000 | 0.590 | 1.960 | 0.500 |
| Buteo buteo | 0.385 | 0.250 | 1.770 | 0.500 |
| Buteo hemilasius | 0.714 | 0.429 | 1.880 | 0.500 |
| Buteo lagopus | 0.244 | 0.250 | 1.680 | 0.500 |
| Ciconia boyciana | 0.201 | 0.250 | 1.530 | 0.500 |
| Ciconia nigra | 1.000 | 0.560 | 1.940 | 0.500 |
| Circus aeruginosus | 1.000 | 0.560 | 1.940 | 0.500 |
| Circus cyaneus | 0.233 | 0.250 | 1.640 | 0.500 |
| Circus melanoleucos | 0.230 | 0.250 | 1.630 | 0.500 |
| Circus spilonotus | 0.116 | 0.250 | 1.230 | 0.500 |
| Clanga clanga | 1.000 | 0.560 | 1.940 | 0.500 |
| Falco peregrinus | 1.000 | 0.575 | 1.950 | 0.500 |
| Falco rusticolus | 1.000 | 0.605 | 1.970 | 0.500 |
| Falco tinnunculus | 0.136 | 0.250 | 1.120 | 0.500 |
| Grus japonensis | 0.050 | 0.250 | 1.000 | 0.500 |
| Grus leucogeranus | 0.288 | 0.250 | 1.720 | 0.500 |
| Grus monacha | 0.481 | 0.250 | 1.820 | 0.500 |
| Grus vipio | 0.207 | 0.250 | 1.550 | 0.500 |
| Mergus squamatus | 0.800 | 0.500 | 1.900 | 0.500 |
| Numenius minutus | 0.586 | 0.321 | 1.850 | 0.500 |
| Otis tarda | 0.236 | 0.250 | 1.650 | 0.500 |
| Pandion haliaetus | 1.000 | 0.515 | 1.910 | 0.500 |
| Platalea leucorodia | 1.000 | 0.515 | 1.910 | 0.500 |
| Platalea minor | 1.000 | 0.605 | 1.970 | 0.500 |
| Strix uralensis | 1.000 | 0.560 | 1.940 | 0.500 |

| 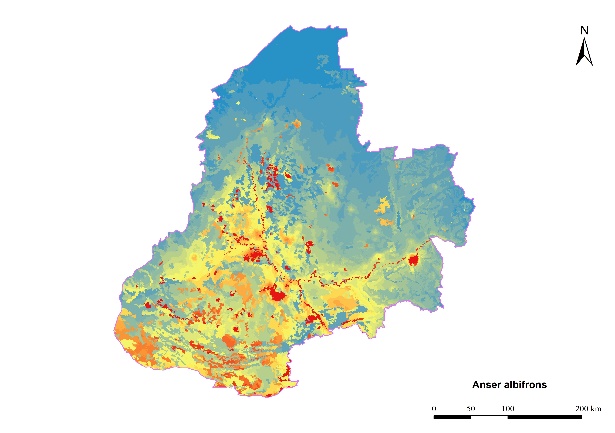 | 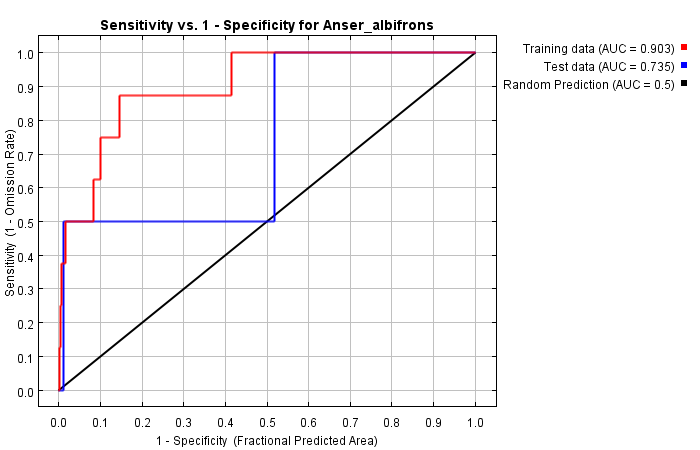 |
| --- | --- |
| 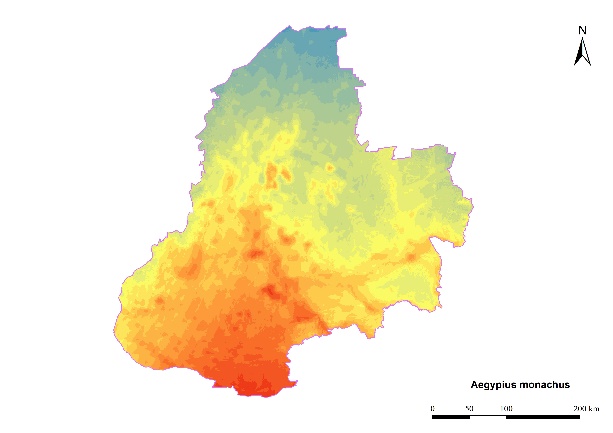 | 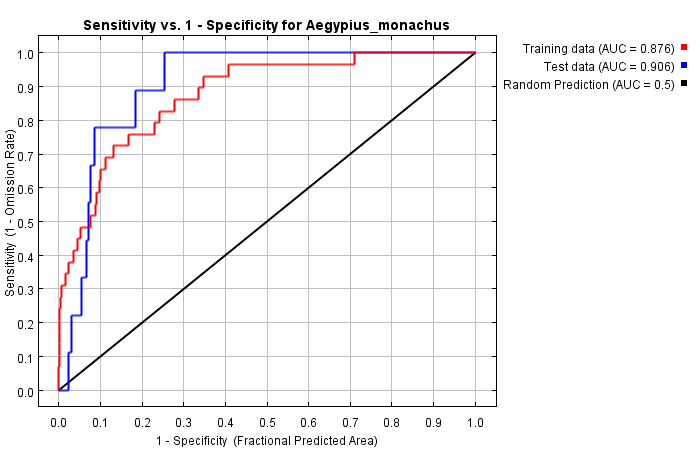 |
| 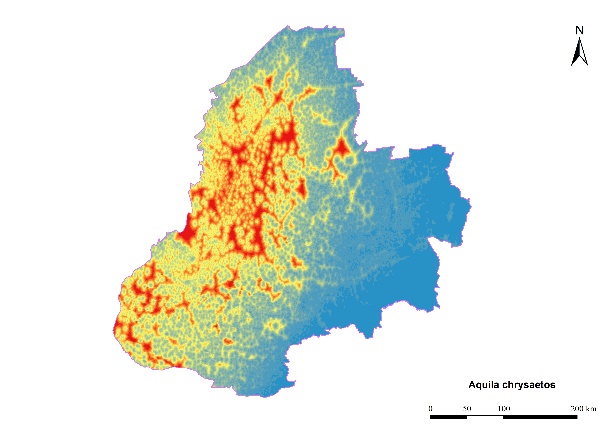 | 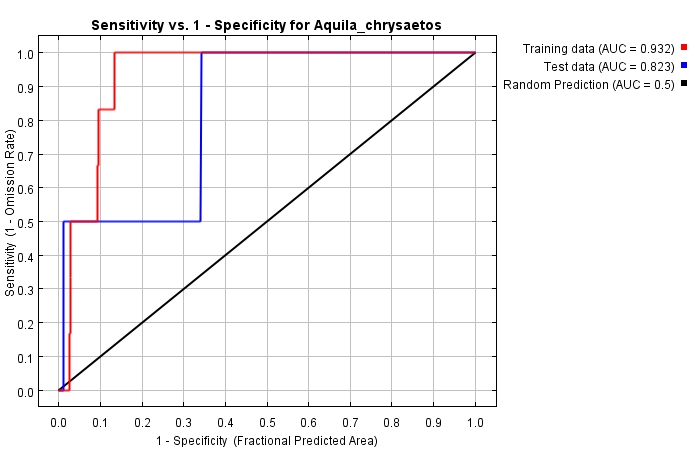 |
| 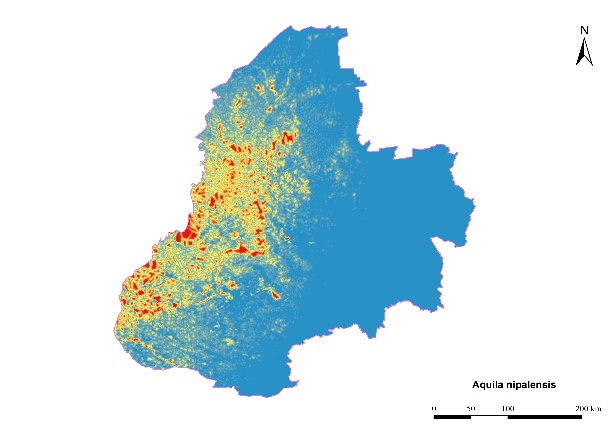 | 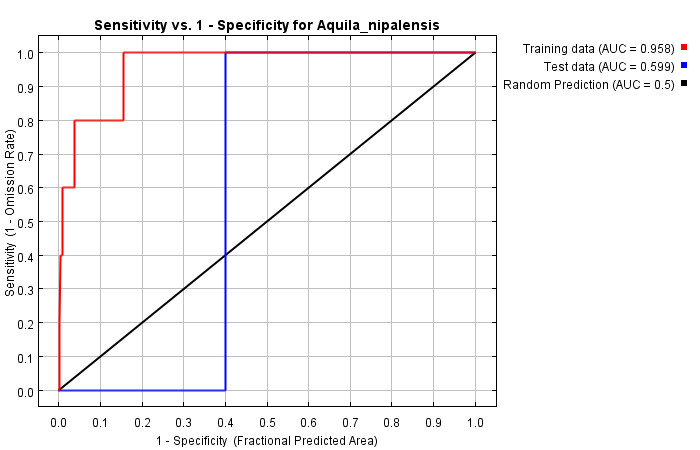 |
| 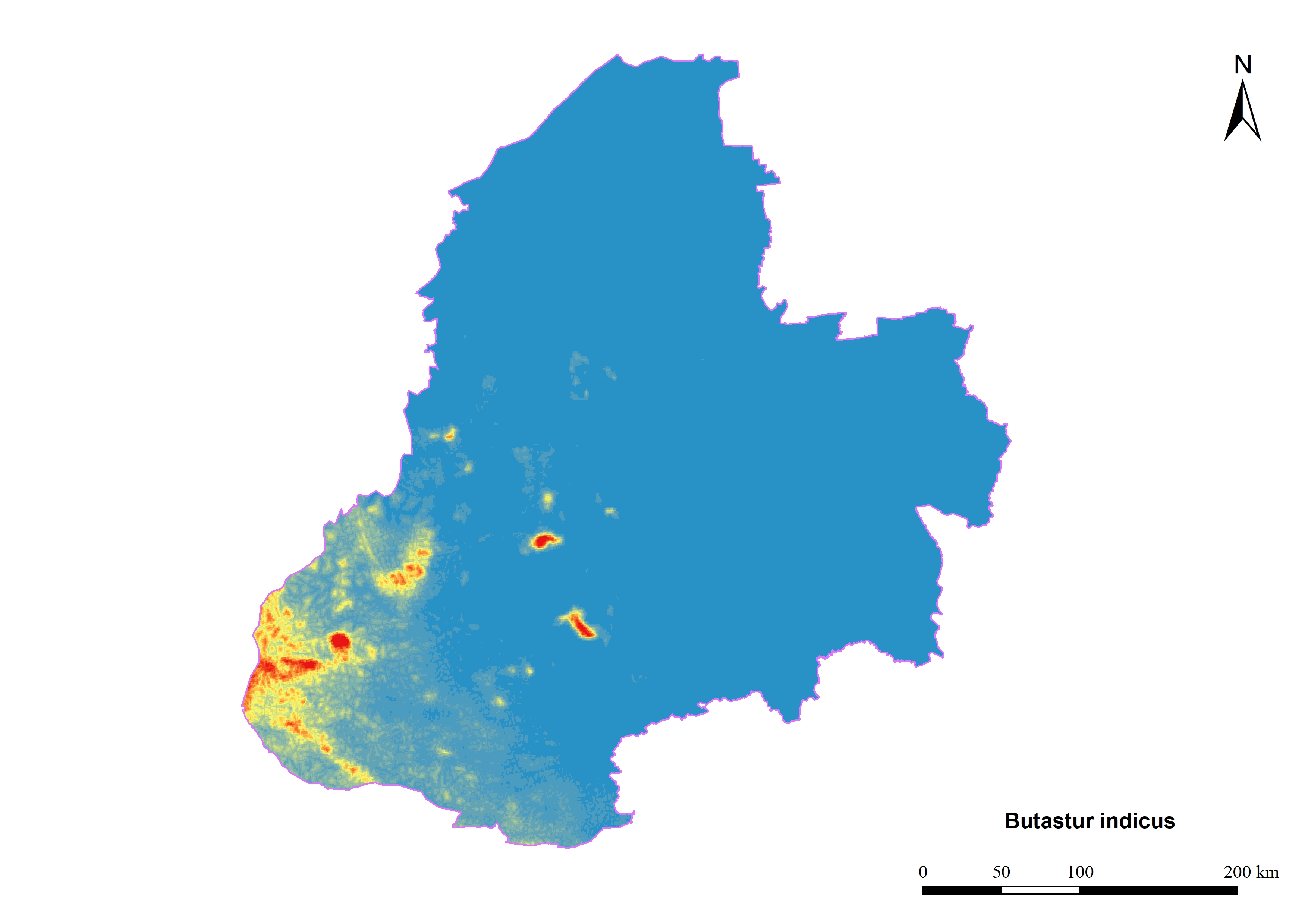 | 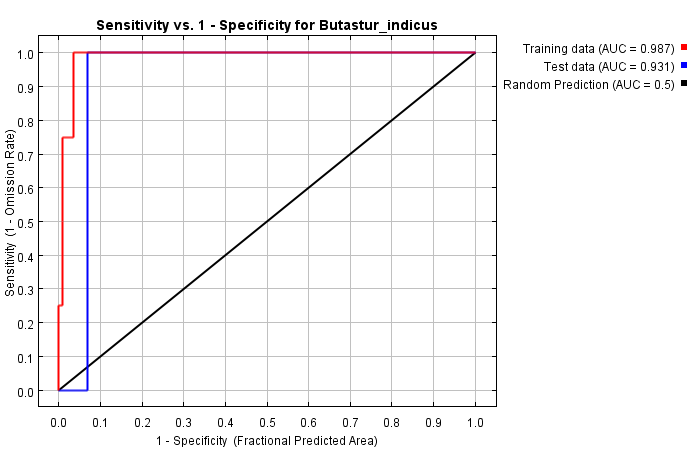 |
| 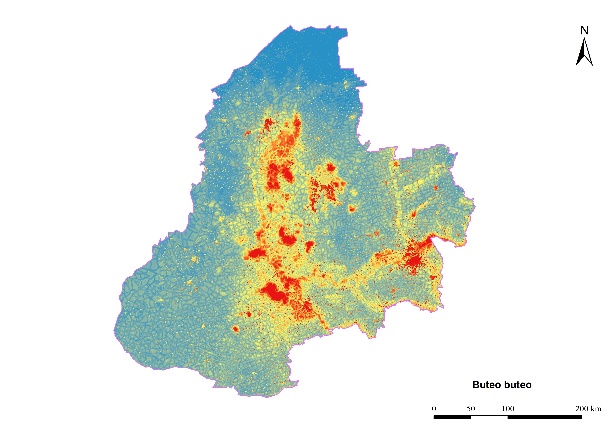 | 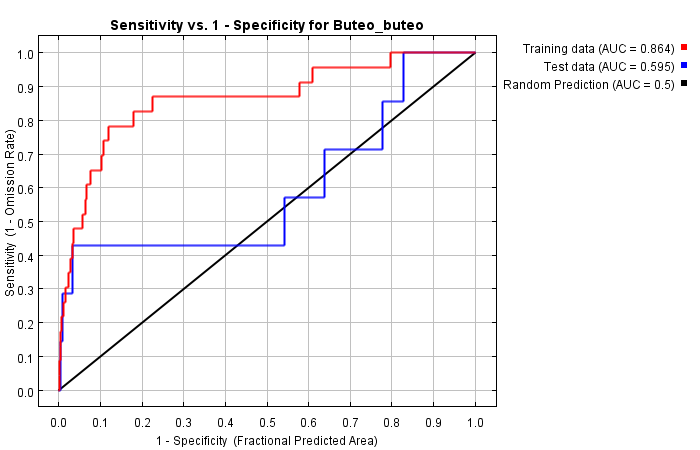 |
| 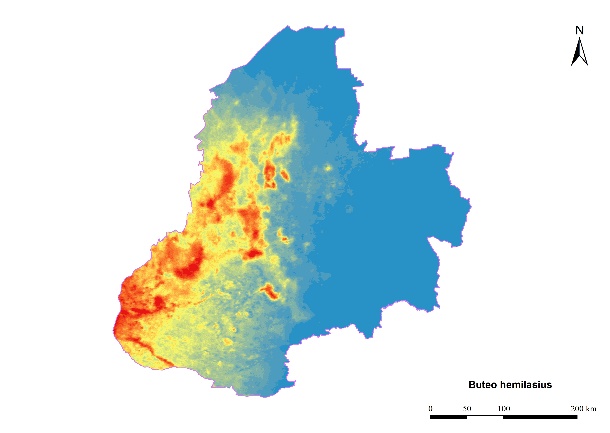 | 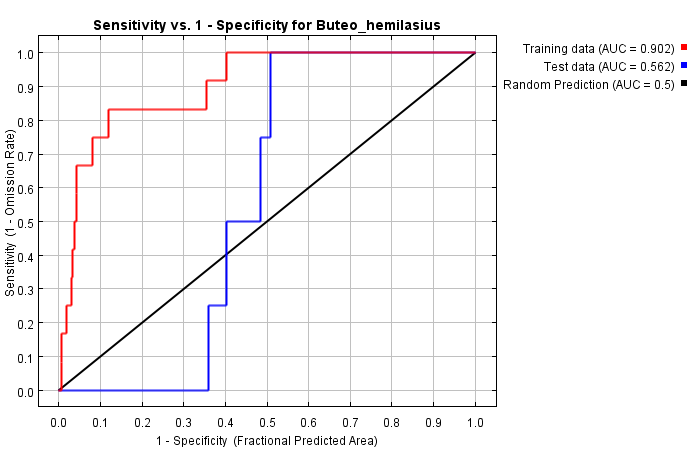 |
| 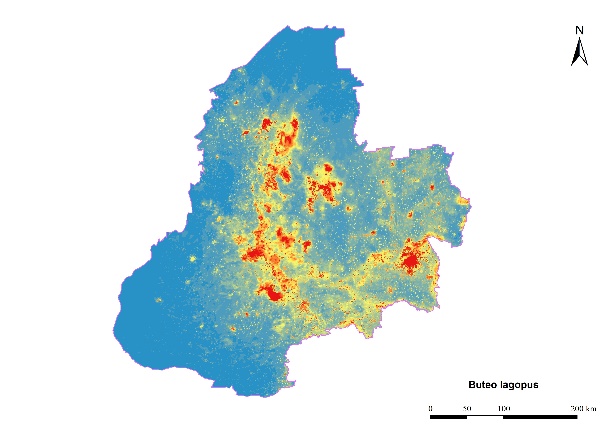 | 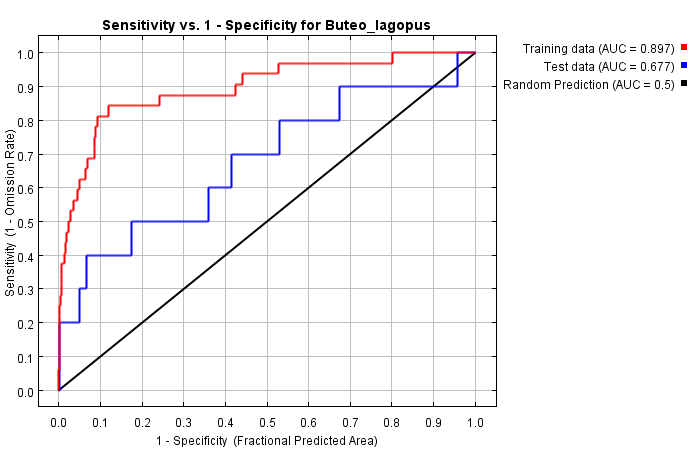 |
| 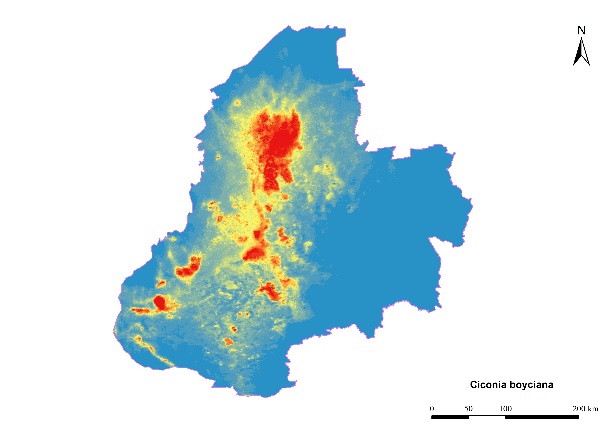 | 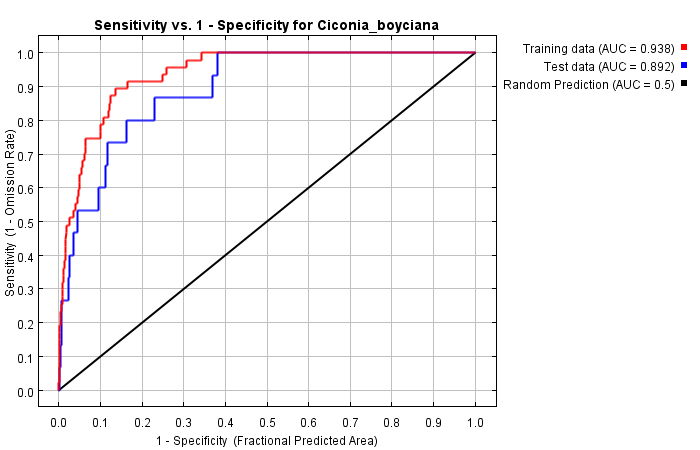 |
| 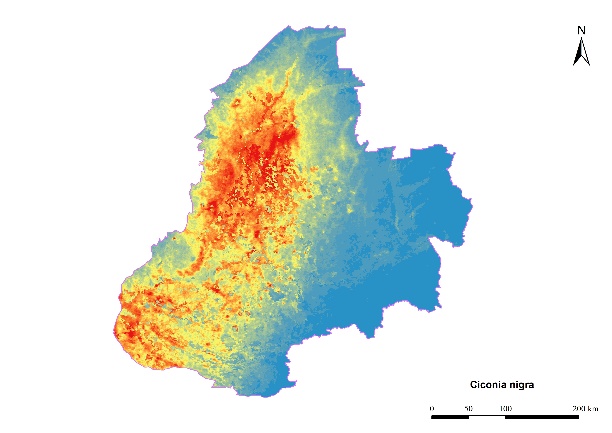 | 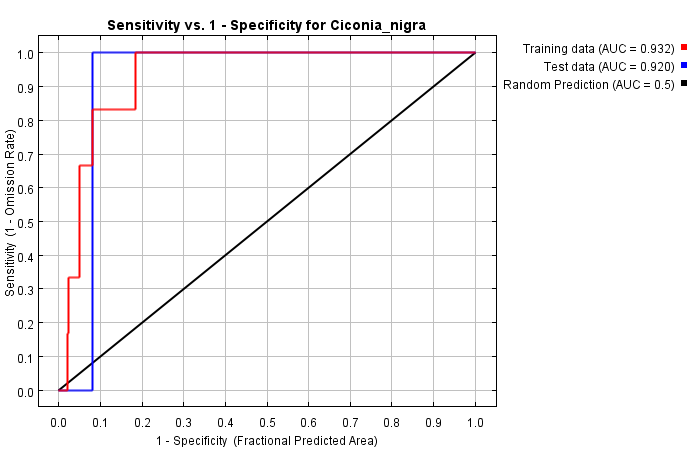 |
| 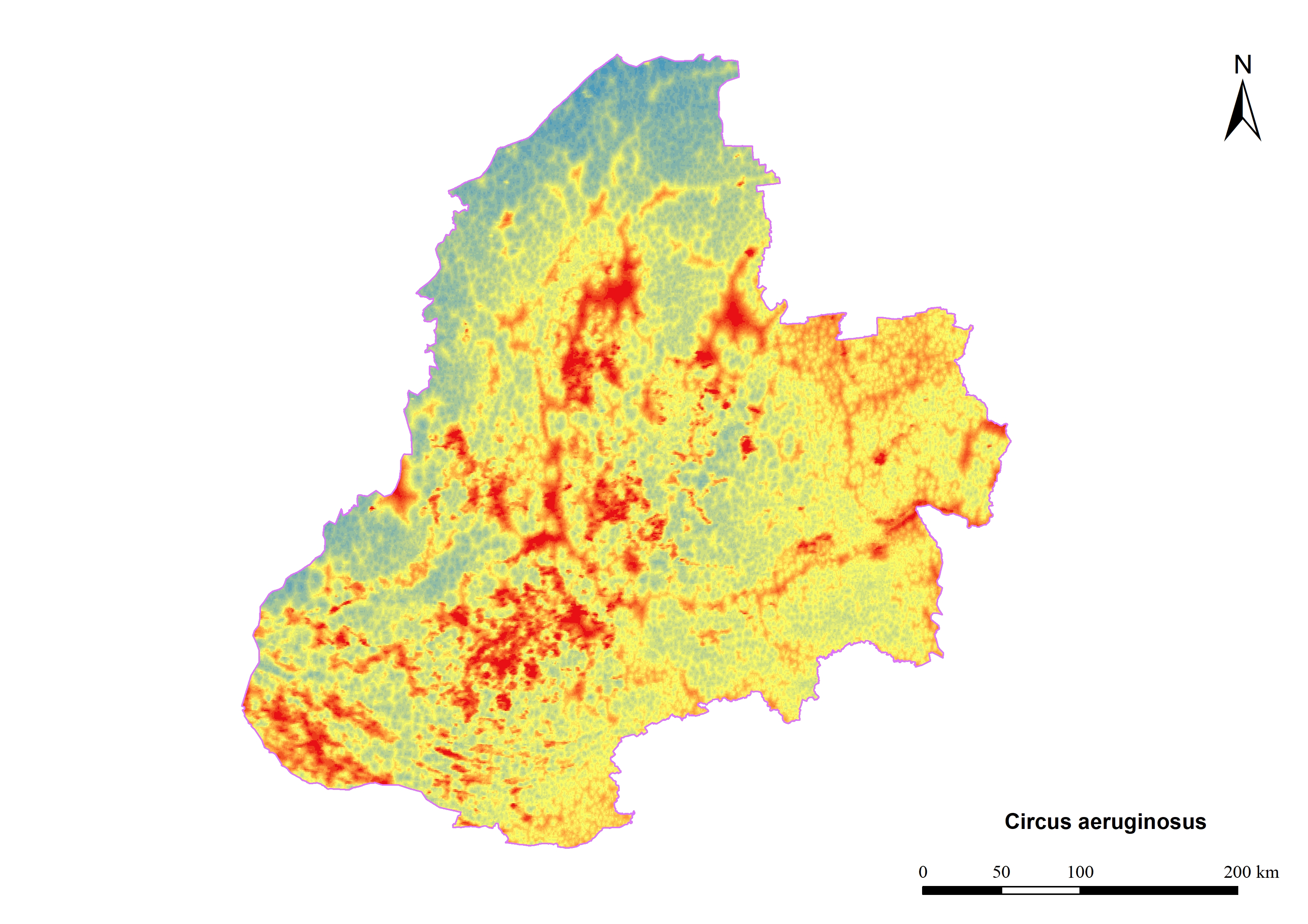 | 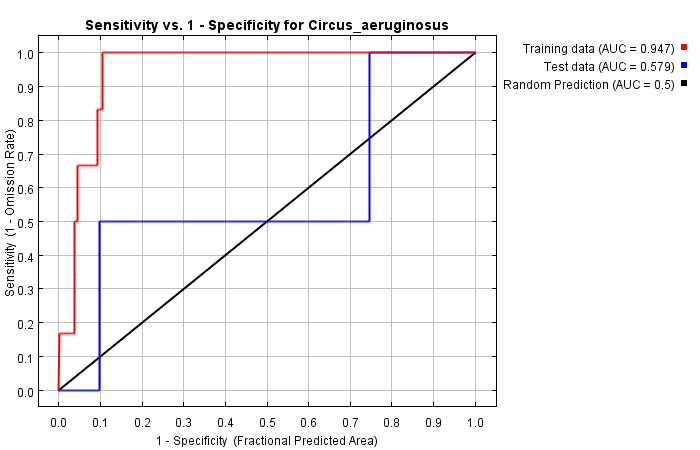 |
| 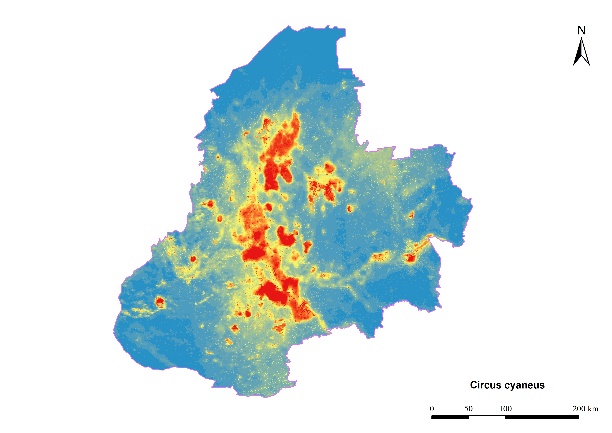 | 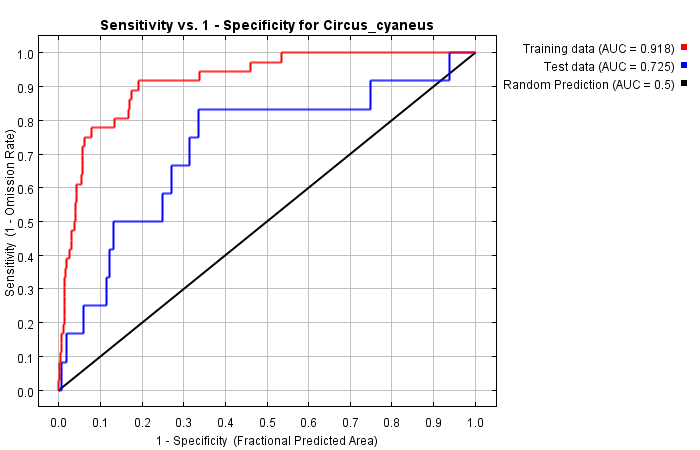 |
| 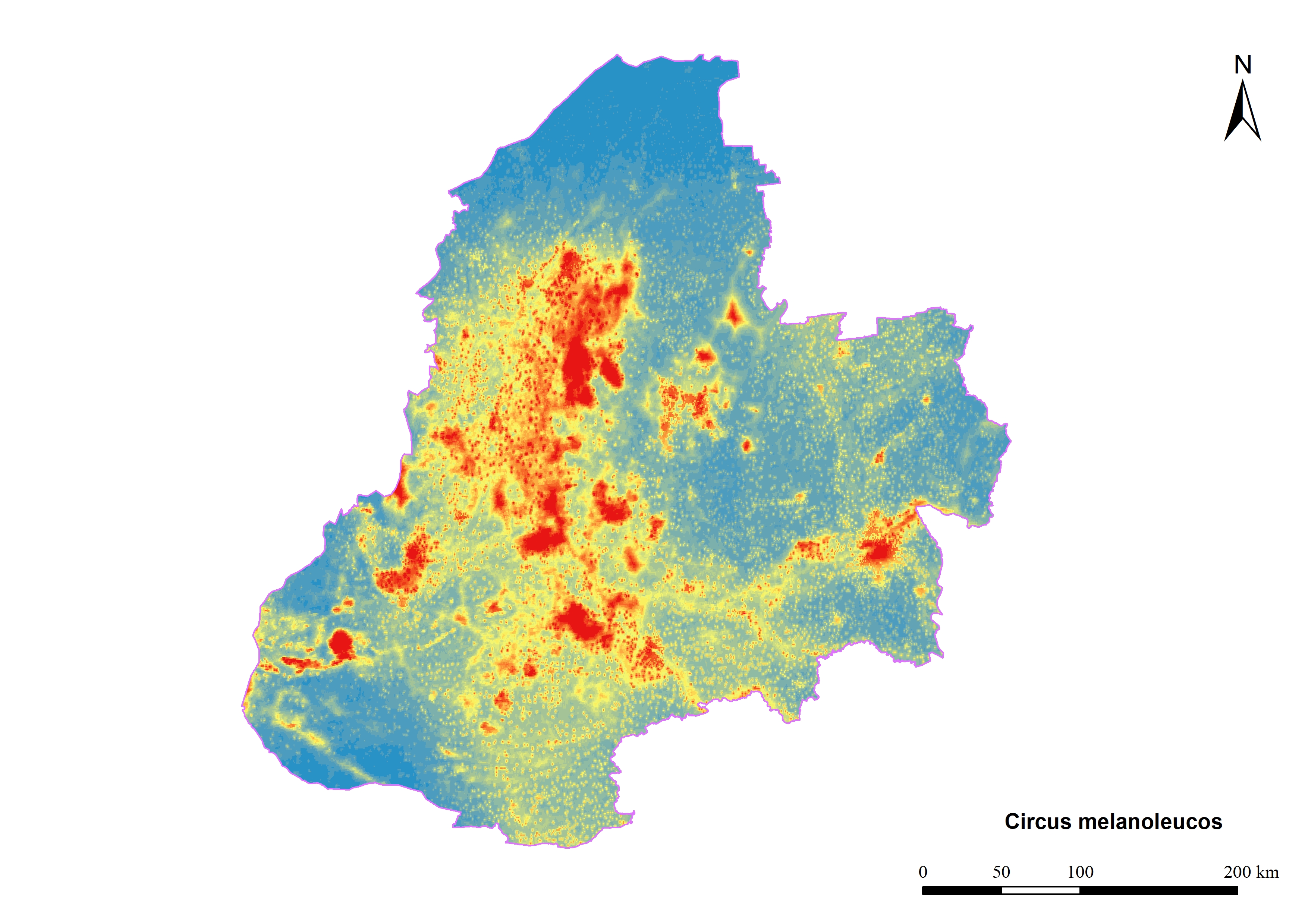 | 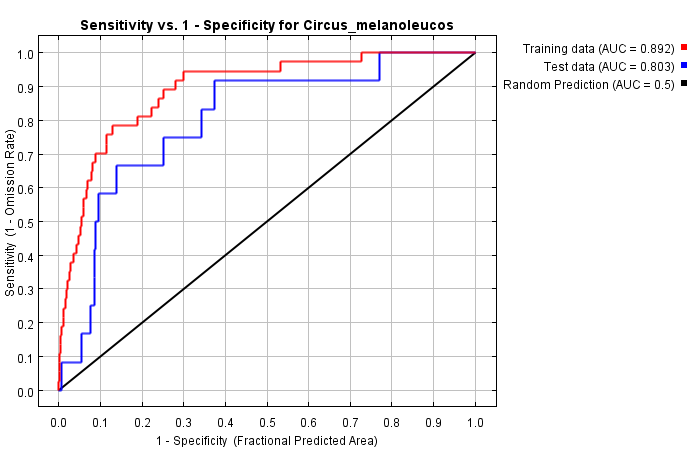 |
| 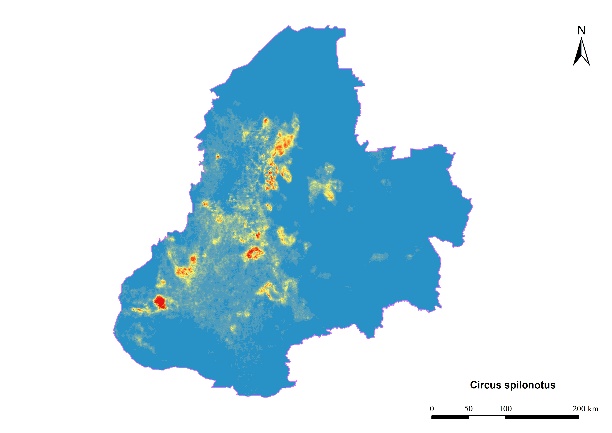 | 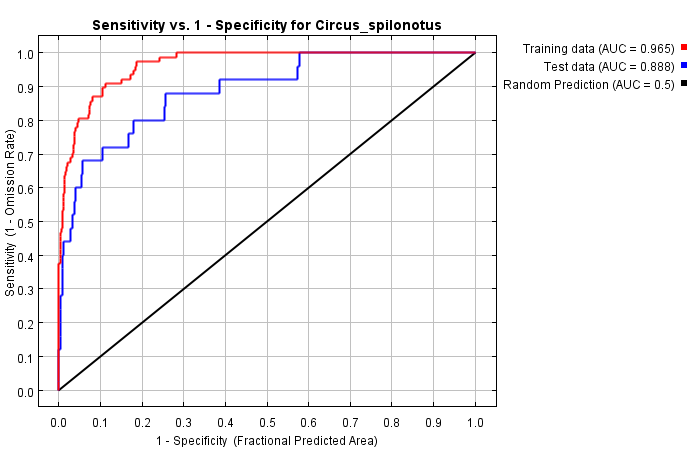 |
| 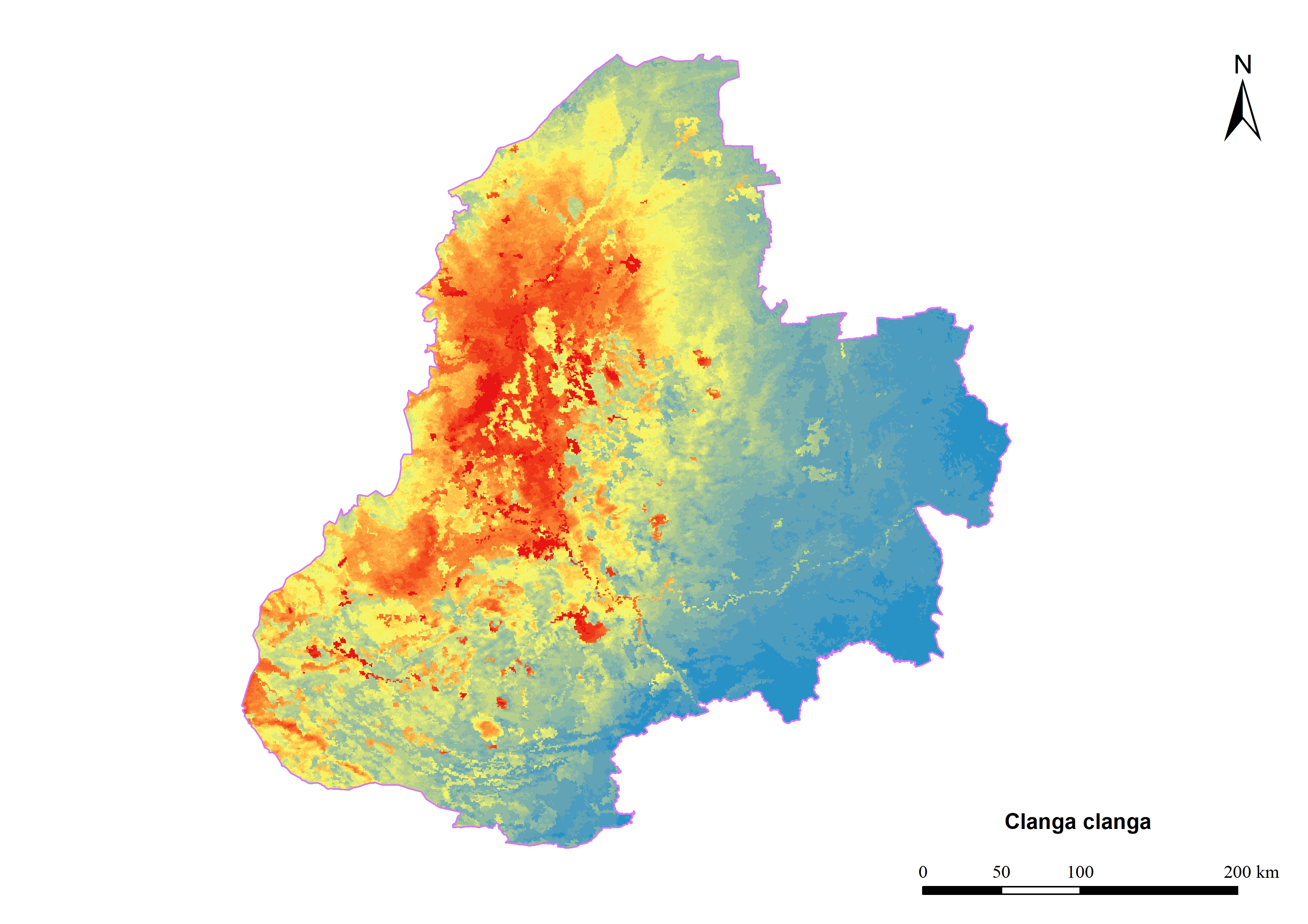 | 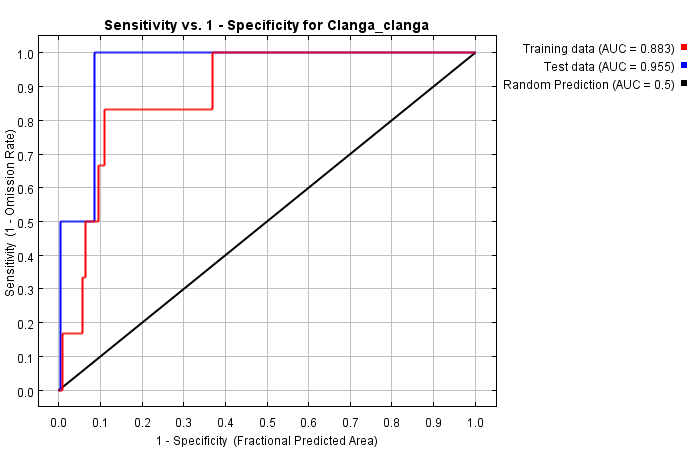 |
| 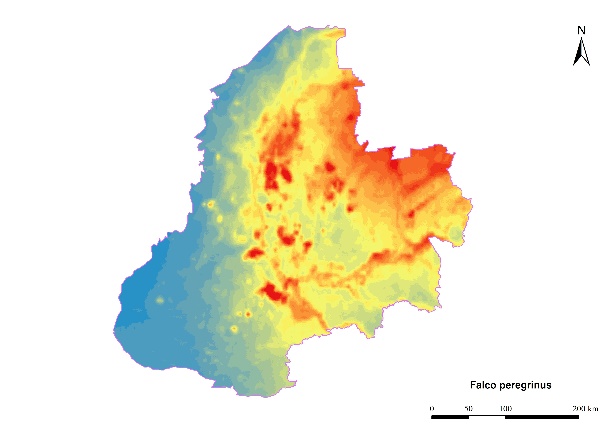 | 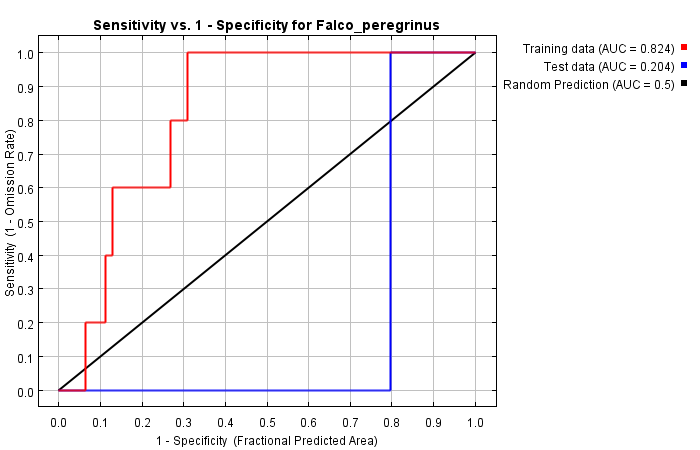 |
| 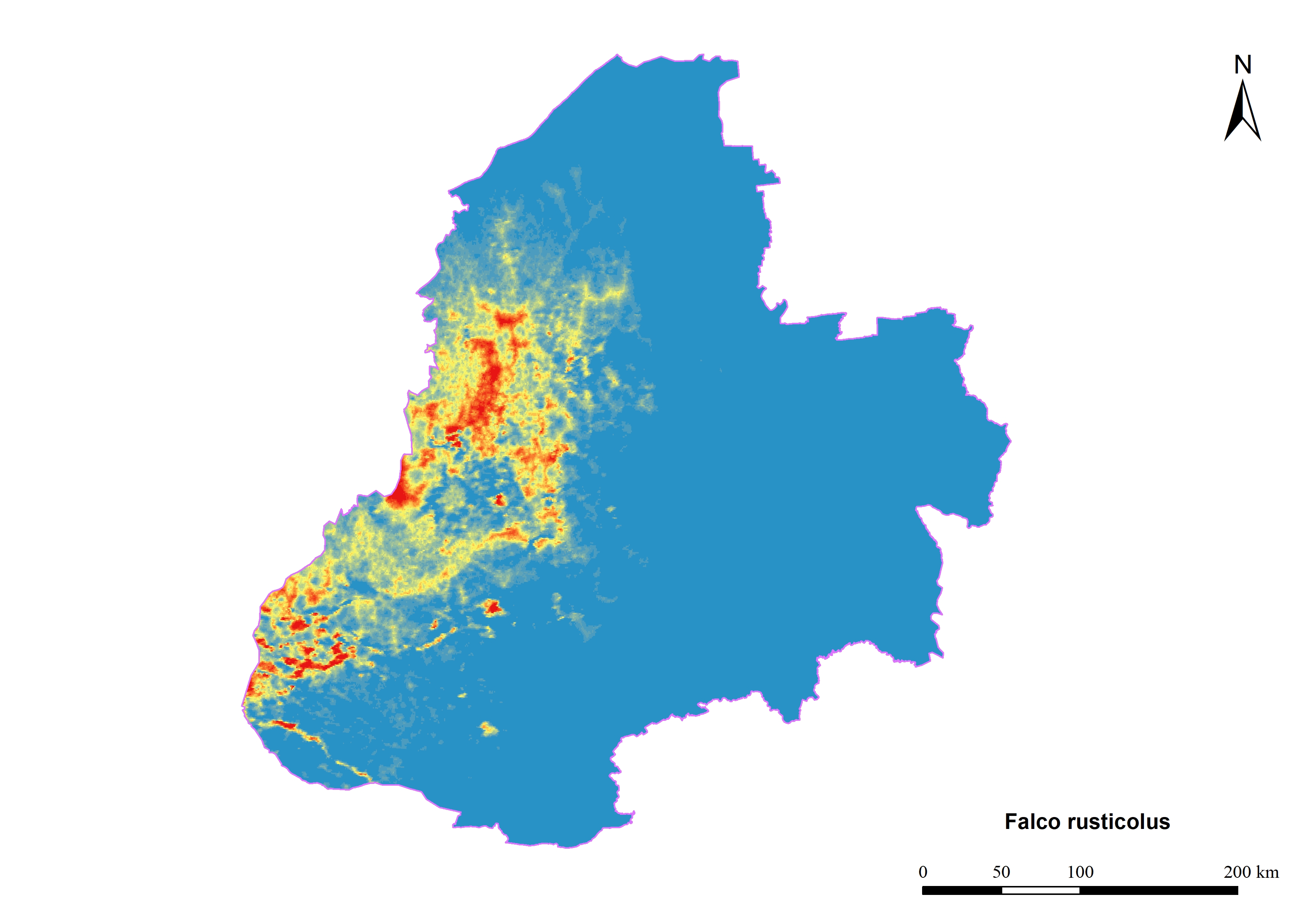 | 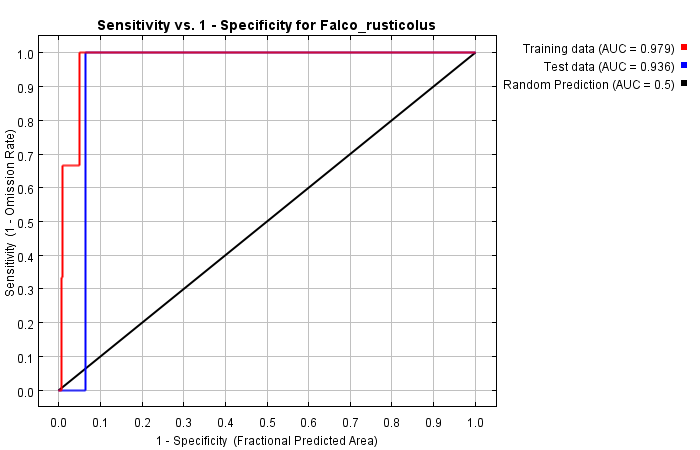 |
| 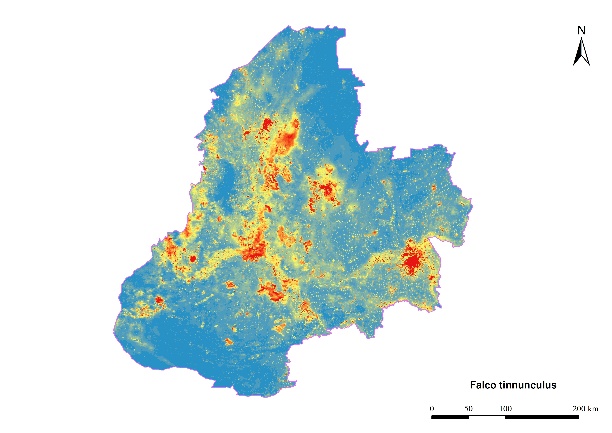 | 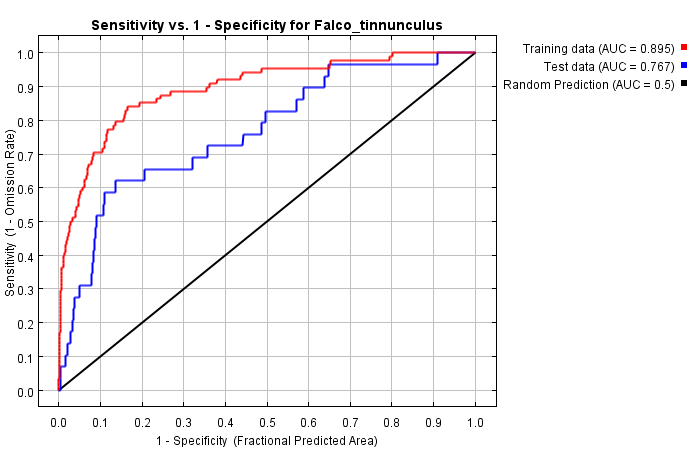 |
| 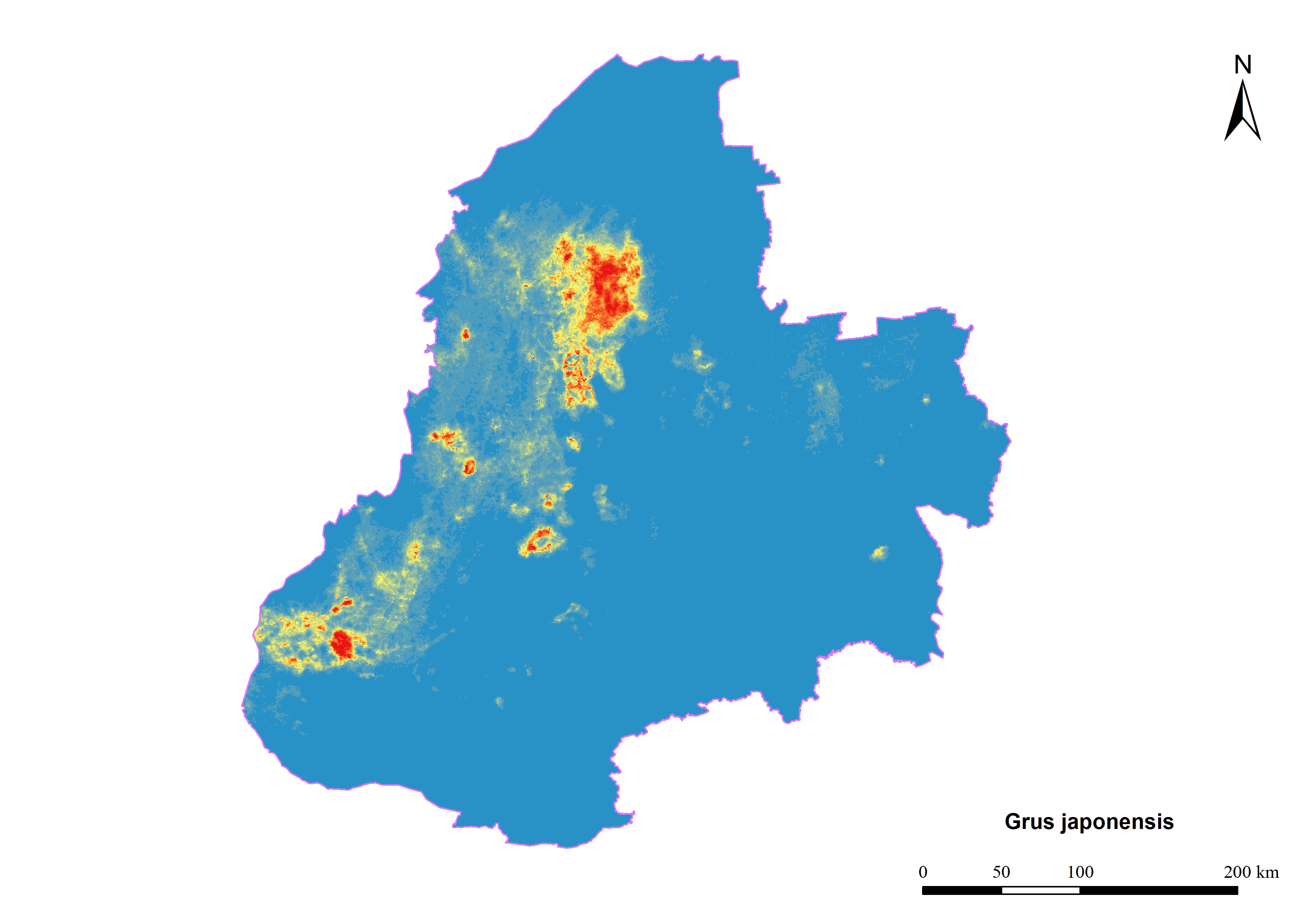 | 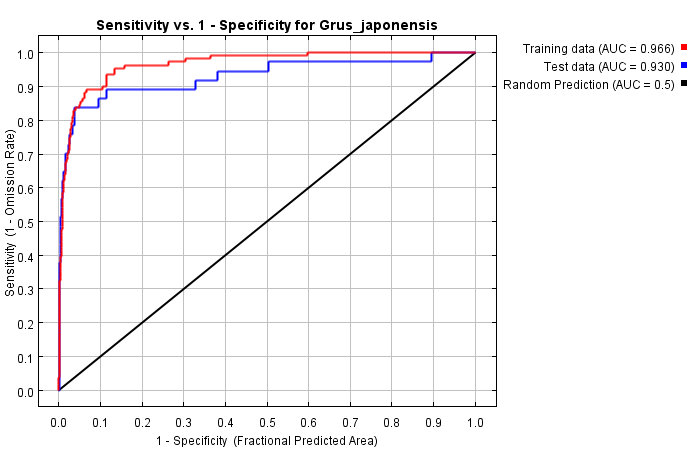 |
| 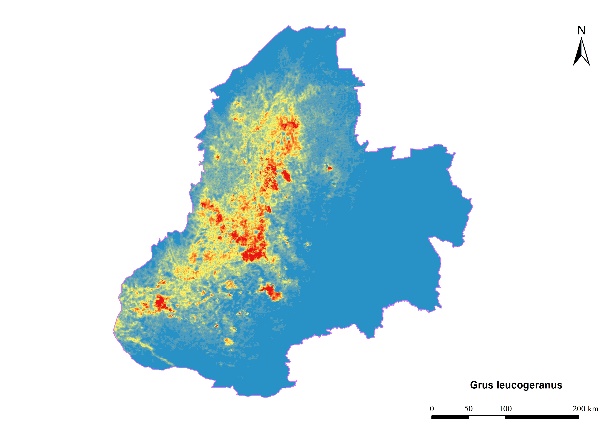 | 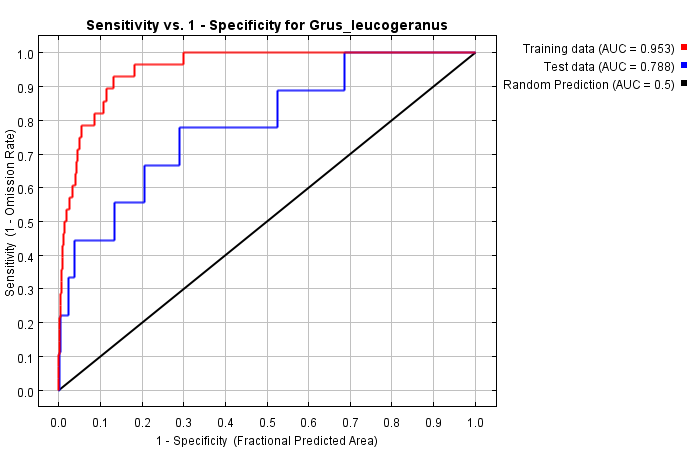 |
| 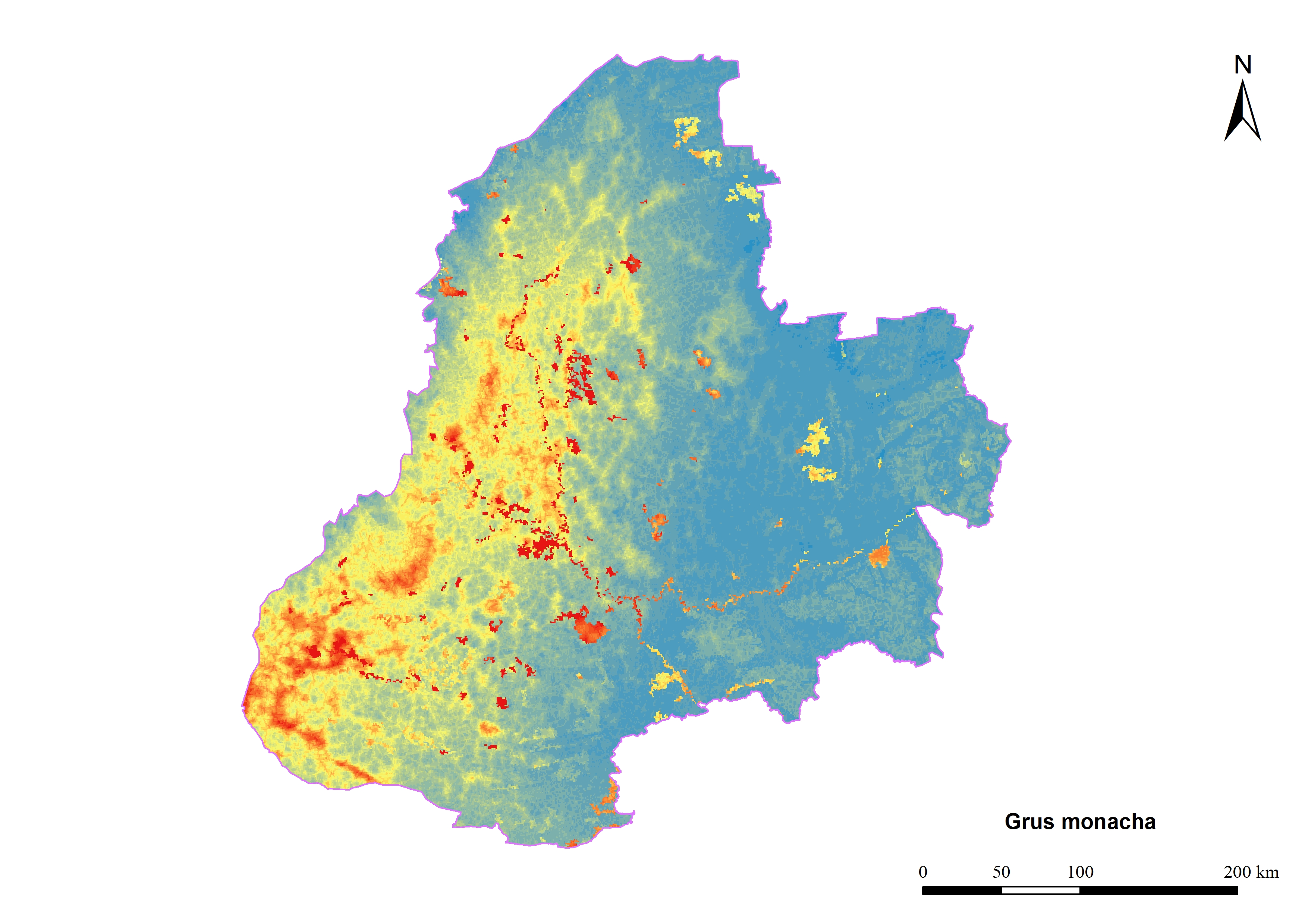 | 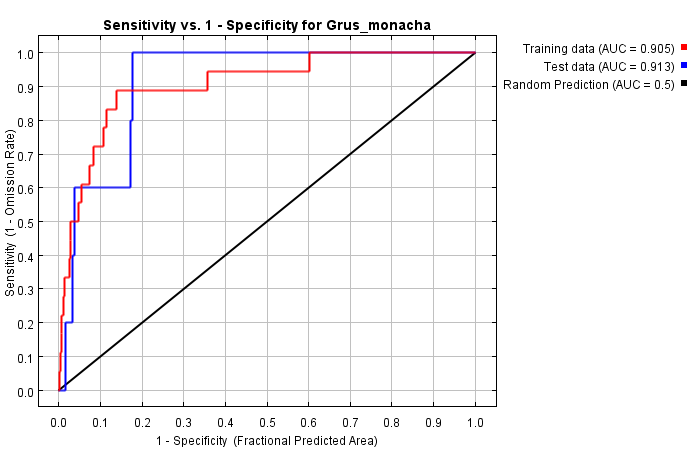 |
| 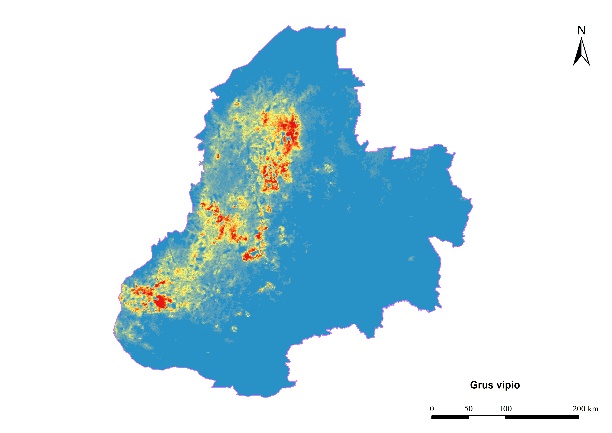 | 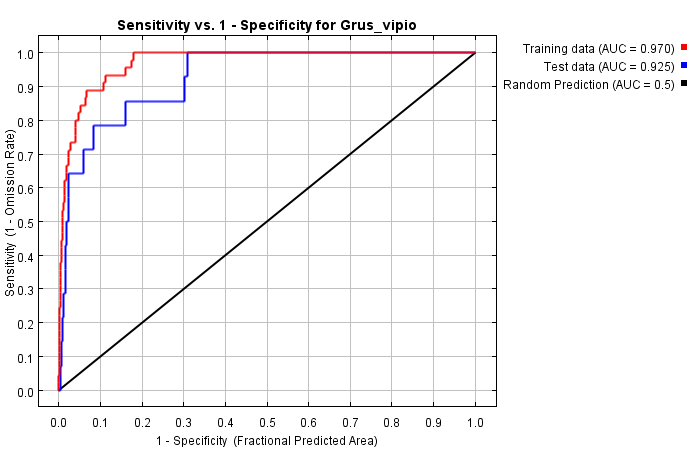 |
| 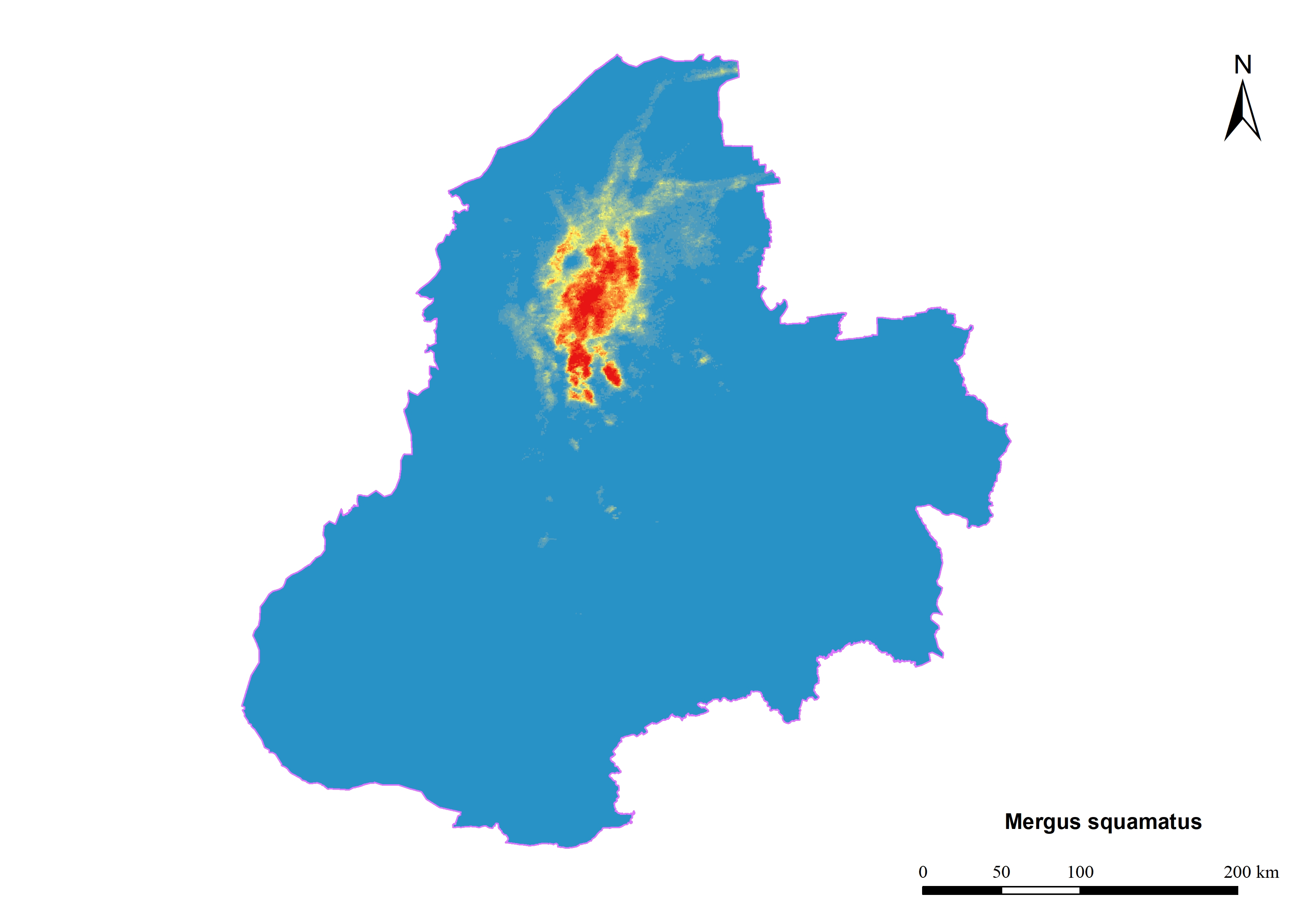 | 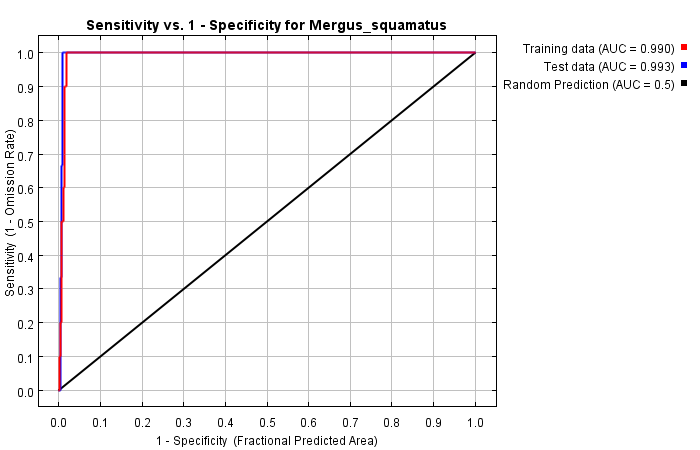 |
| 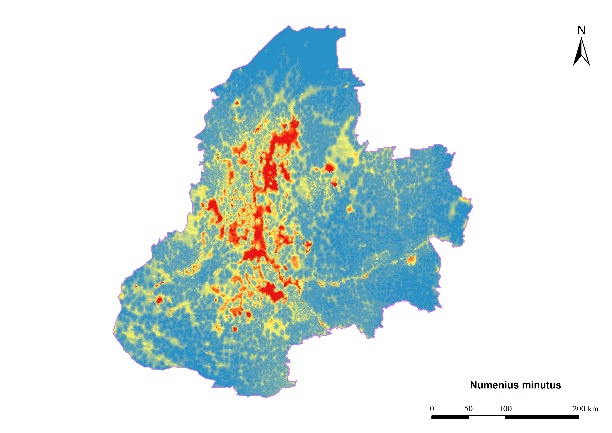 | 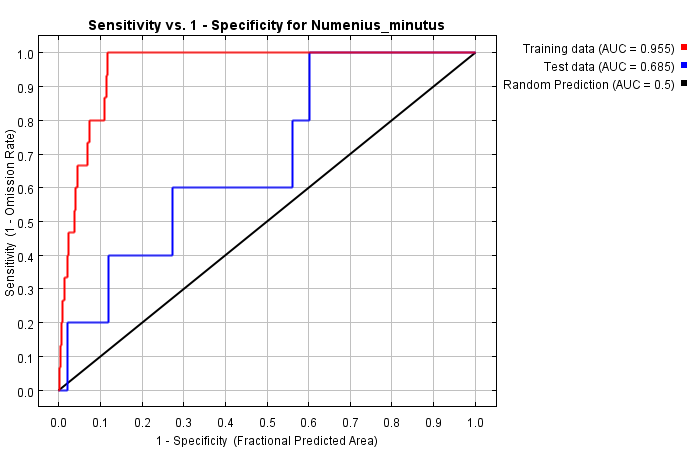 |
| 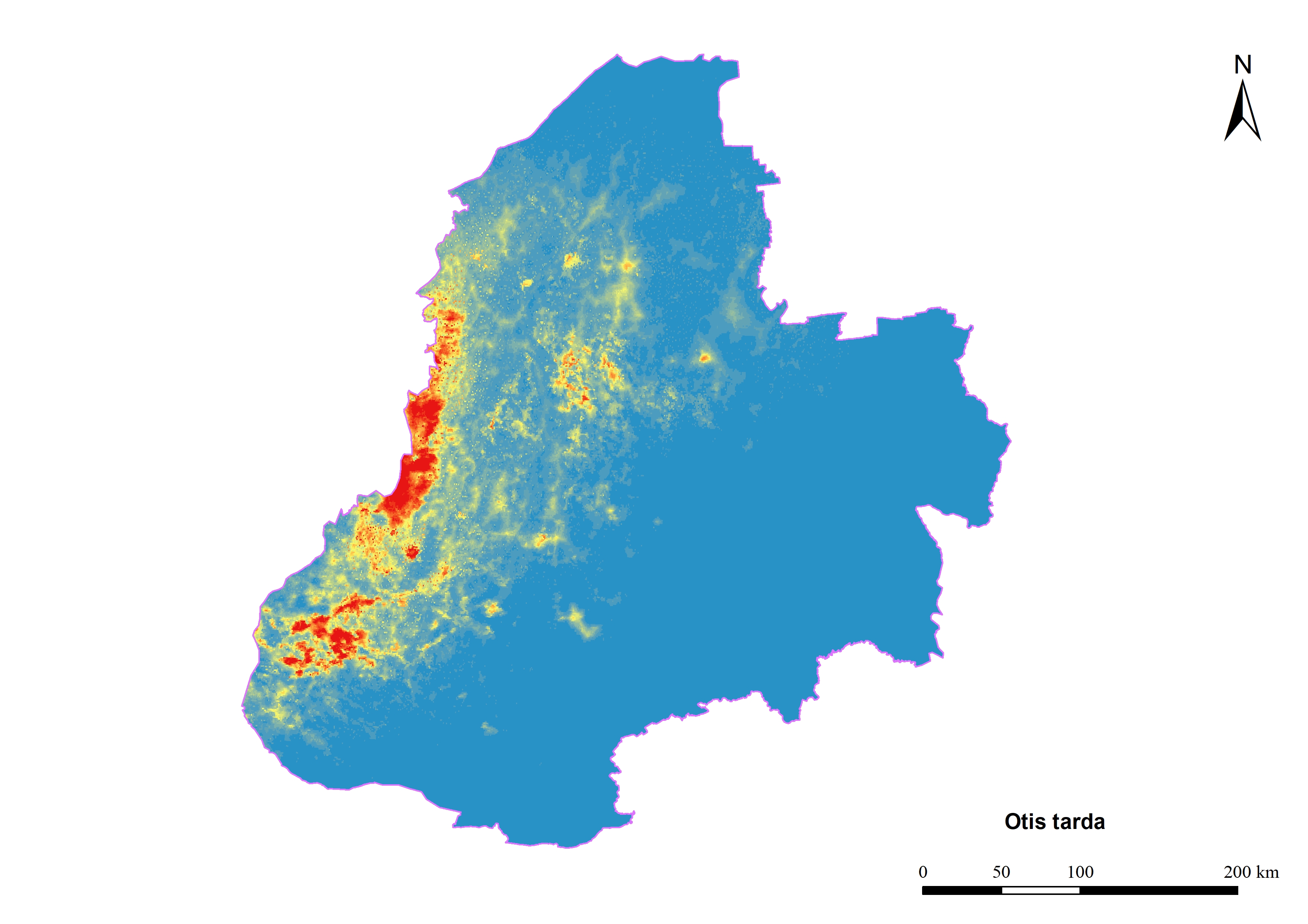 | 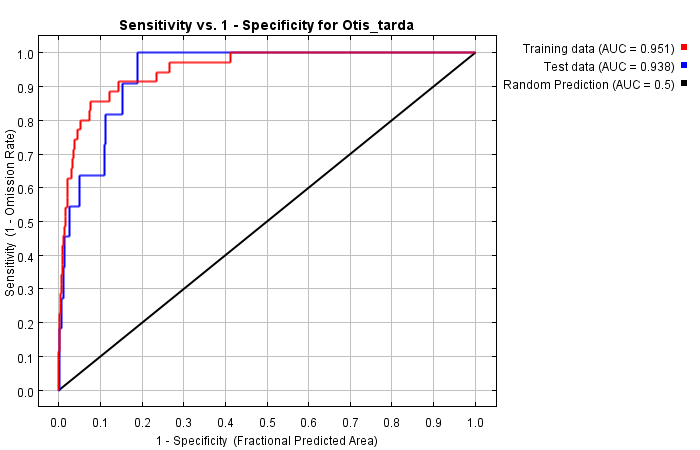 |
| 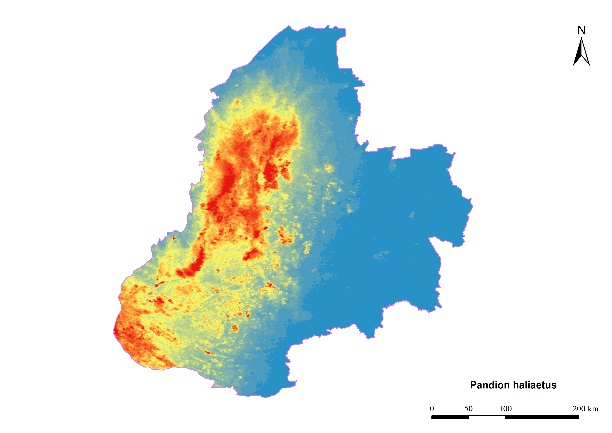 | 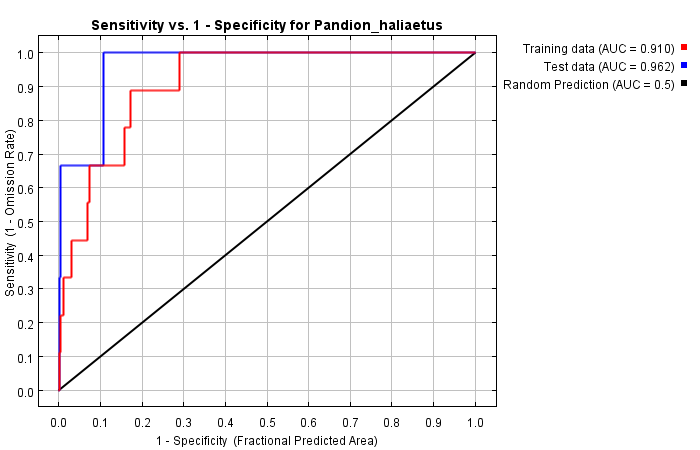 |
| 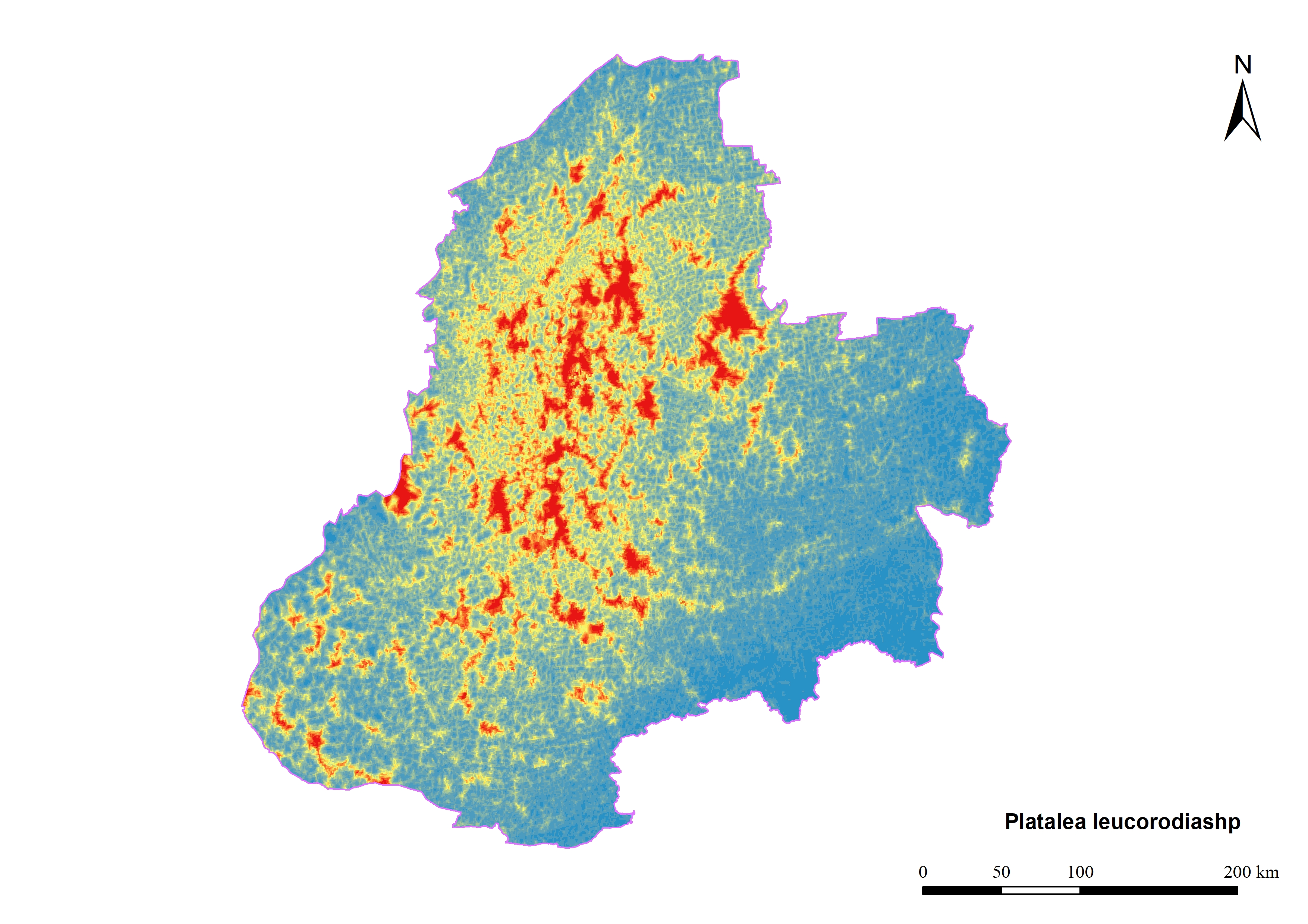 | 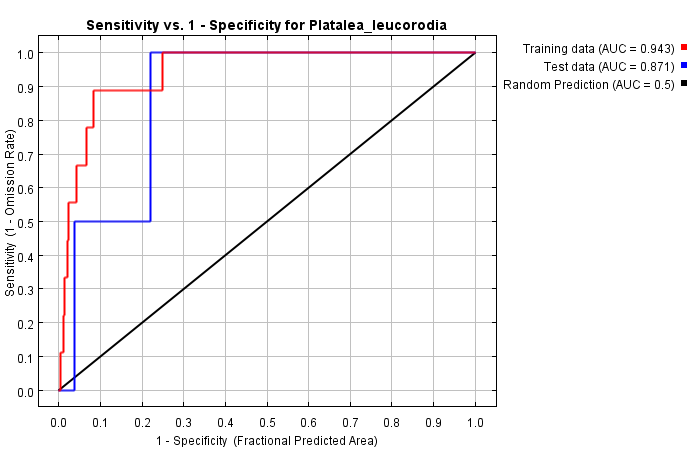 |
| 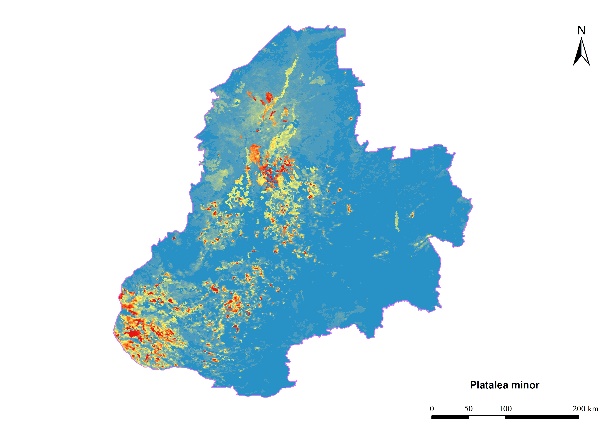 | 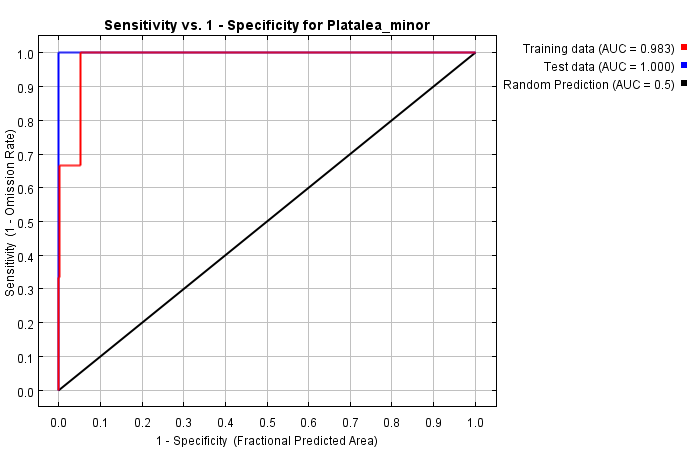 |
| 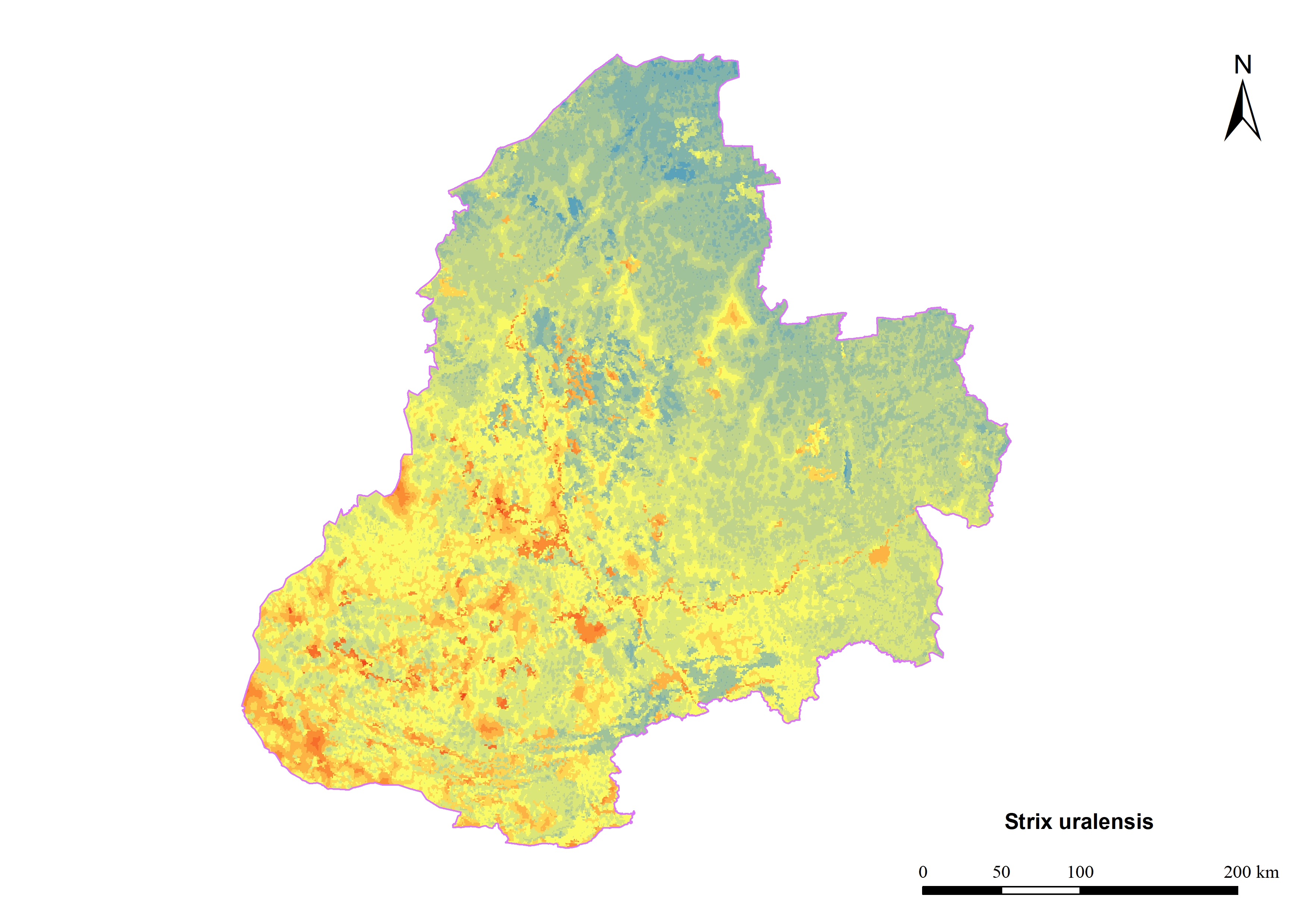 | 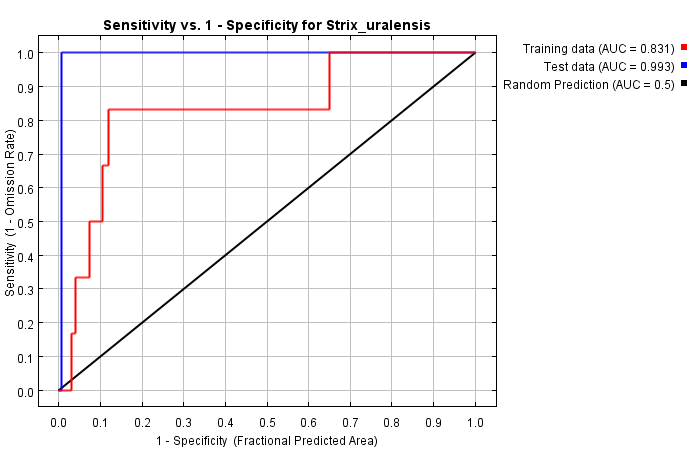 |
| **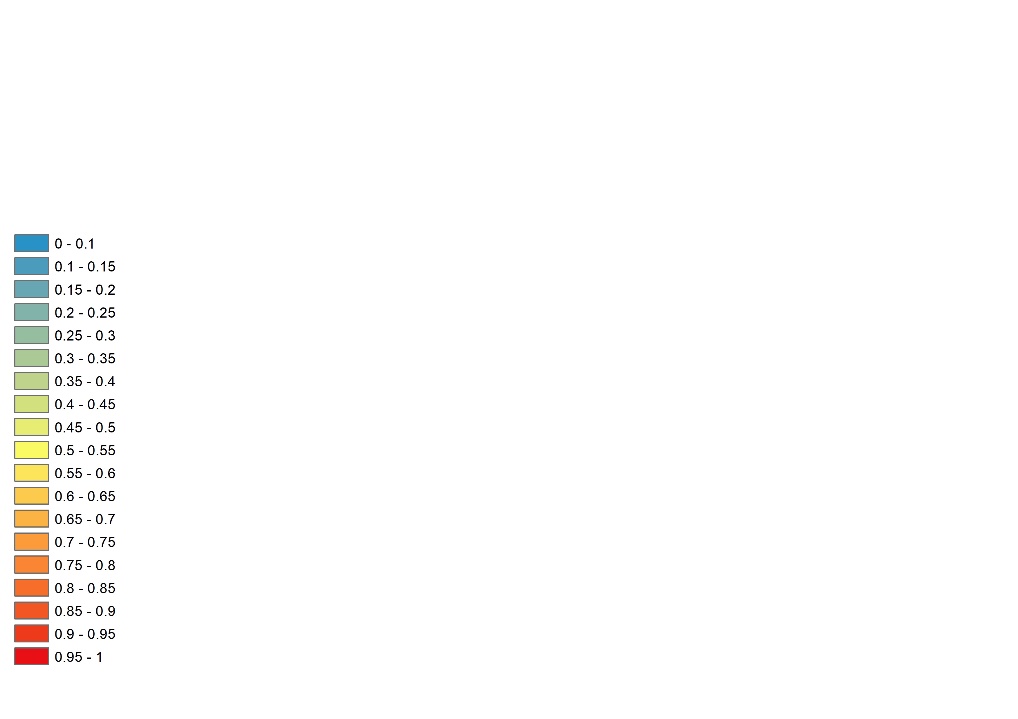** |  |

**Figure S2** The predicted probabiltiy of occurrrnce of each of 29 species in the Songnen Plain. The final consensus probability map for each species is a sum of output from ten replicates distribution models. All maps symbologies are recouloured to match the same legend


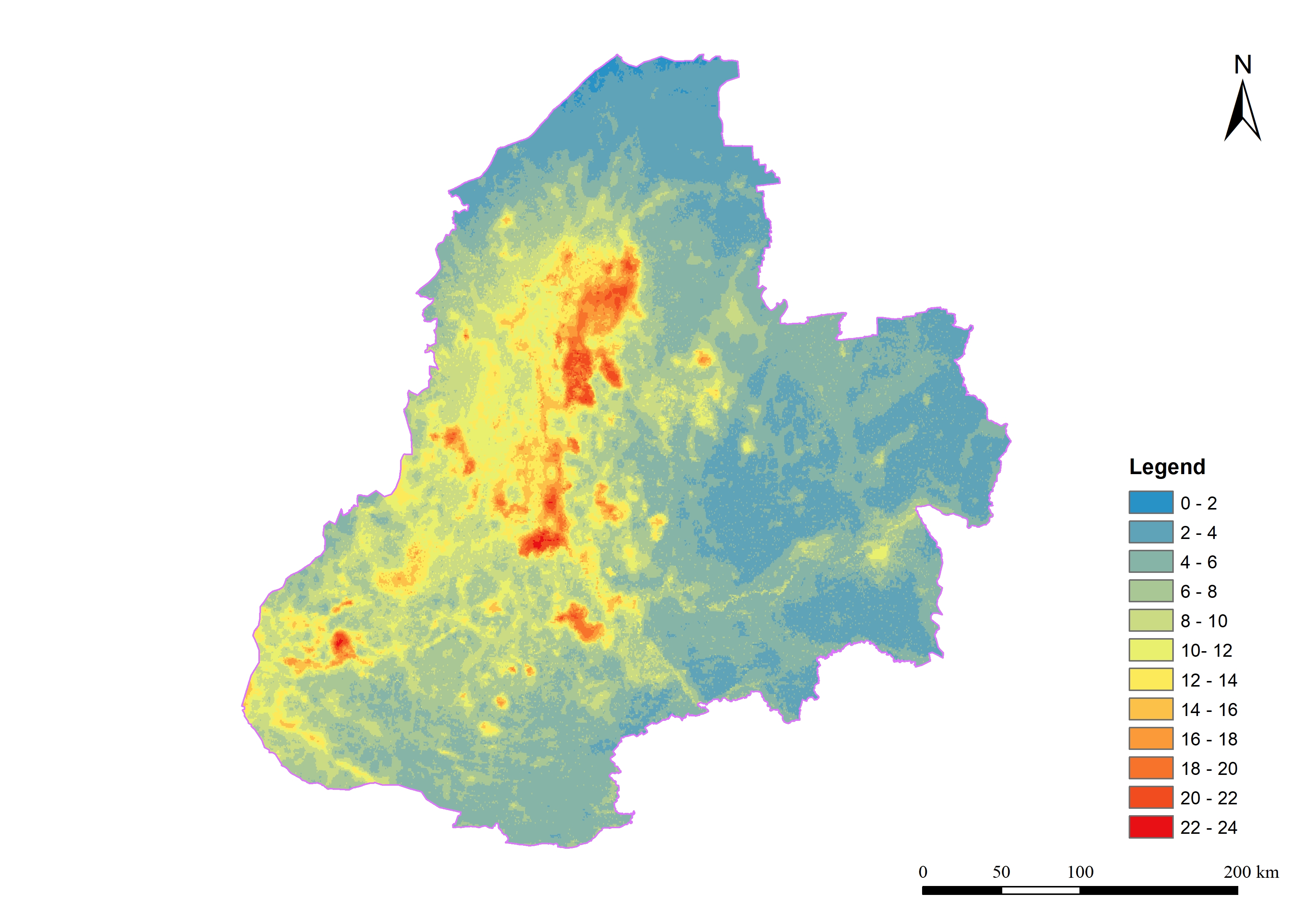


**Figure S3** Predicted endangered species richness for the Songnen Plain, based on summed habitat suitabilities from individual distribution models for 29 species

**Table S6** The sensitivity of different land use and land cover to different threats

| Code | Type | Habitat suitability | Threat sources | | | | | |
| --- | --- | --- | --- | --- | --- | --- | --- | --- |
|  |  |  | Field | Towns | Villages and Towns | Roads | Railways | Mining sites |
| 1 | Field | 0.3 | 0 | 0.4 | 0.5 | 0.1 | 0.1 | 0.3 |
| 2 | Woodland | 1 | 0.8 | 1 | 1 | 0.6 | 0.5 | 1 |
| 3 | Grassland | 0.9 | 0.7 | 0.5 | 0.5 | 0.6 | 0.3 | 0.8 |
| 4 | Wetlands | 1 | 0.9 | 0.8 | 0.9 | 0.6 | 0.6 | 1 |
| 5 | Water area | 1 | 0.6 | 0.7 | 0.7 | 0.5 | 0.5 | 0.8 |
| 6 | Built-up area | 0 | 0 | 0 | 0 | 0 | 0 | 0 |
| 7 | Unused land | 0.5 | 0 | 0 | 0 | 0 | 0 | 0 |
